# Formation of multiple complex light structures simultaneously in 3D volume using a single binary phase mask

**DOI:** 10.1038/s41598-023-42087-0

**Published:** 2023-10-07

**Authors:** Amit Kumar, Sarvesh Thakur, S. K. Biswas

**Affiliations:** Bio-NanoPhotonics Laboratory, Department of Physical Sciences, Indian Institute of Science Education and Research-Mohali, Knowledge City, Sector 81, SAS Nagar, Manauli, PO 140306 India

**Keywords:** Adaptive optics, Displays, Biophotonics, Optics and photonics, Applied optics, Optoelectronic devices and components

## Abstract

Complex structure formation inside or through turbid media is a challenging task due to refractive index inhomogeneity, random light scattering, and speckle noise formation. In this article, we have coupled the data regression model in the R-squared metric and used its advantages as a fitness function in the genetic algorithm to advance the resolution and structural uniformity. As a compatible system with the binary genetic algorithm, we have presented a cost-effective iterative wavefront shaping system-design with binary phase modulation using an affordable ferroelectric liquid crystal (FLC) based binary-phase spatial light modulator (SLM). R-squared metric in the genetic algorithm is analyzed to optimize the binary phase mask, and the prototype system based on iterative binary phase modulation has been validated with a 120-grit ground glass diffuser and fresh chicken tissues of thickness 307 ${\upmu }\text {m}$ and 812 ${\upmu }\text {m}$. The detailed results show that the proposed cost-effective wavefront shaping system with data regression model assisted R-squared fitness function can construct high-resolution multiple complex hetero-structures simultaneously in 3D volume using an optimized single phase-mask.

## Introduction

Scattering of light in living or non-living tissue and other disordered media is one of the primary challenges in biomedical imaging, deep tissue imaging, biomedical engineering, and is an active research area in the adaptive optics and bio-engineering community^1–9^. Focusing light through scattering media such as tissue has many applications in structural light illumination microscopy^10^, fluorescence imaging^7,11^, live cell imaging, neuron excitation/imaging^12,13^, optical trapping^14^, and holographyic display. Scientists worldwide are currently engaged in addressing the challenges arising from scattering in various types of optical and radiation-based biomedical imaging^1,4–13^. Inhomogeneity of refractive index in the medium, repeated random scattering, and speckle noise due to local interference of light cause an unavoidable distortion of the wavefront^1,4,15^. Modulation of the incident wavefront using spatial light modulator (SLM)^1,4–6^ enables the focusing of light inside or through scattering media, and it has been initially demonstrated experimentally by Vellekoop and Mosk in 2007^16^. Wavefront shaping is mostly performed using an iterative, transmission matrix (TM), and digital optical phase conjugation (DOPC) approach^1,4,16–24^. Recently, it has been observed that evolution-inspired iterative optimization algorithms like genetic algorithm (GA) are well suited for the problem^25–28^. Relevant studies have shown that genetic algorithms perform better in terms of enhancement compared to previously introduced iterative and TM approaches, even in highly noisy environments^25–27^. Feedback-based wavefront shaping techniques have been explored using various iterative computational algorithms and adaptive optics^16,25–27,29–34^. It is also reported in the literature that derivative-free GA^35,36^ based feed back technique is quite effective in neutralizing phase aberration and coping with dynamic noisy environments. A fitness function is used for optimization in iterative feedback-based algorithms to get the desired output. Recently, fitness functions such as target pixels intensity^16,32^, peak-to-background ratio (PBR)^31,37^, Pearson’s correlation coefficient^38,39^, and standard deviation^28^, etc have been reported. The selection of fitness functions to optimize solutions within specific segments involves a trade-off between the advantages and disadvantages of each approach. For example, intensity^16,32^ and PBR^31,37^ based fitness functions are common and have been used widely for focusing light through scattering media. Target intensity based fitness function enhances the intensity at the target spot but shows a noisy background, whereas the PBR based fitness function shows better target intensity and suppressed background intensity^31^. However, both fitness functions are unable to form desired patterns with sufficiently high resolution and structural uniformity^15,39^. A couple of fitness functions have been reported to form different types of light structure in the literature^28,39^.

The formation of structured light through and inside scattering media has several potential applications in the real world problems, such as, in the field of holographic displays^40^, structured light illumination based microscopy^10^, and photolithography. Recently, sequential or temporal 3D holography through scattering media using multiple phase masks has been reported^40–43^. In 2016, Zhuang et al.^41^ demonstrated color imaging through turbid media by considering the memory effect and the point spread function of the optical system. In 2017, Yu et al.^40^ demonstrated the plane-wise projection of dotted patterns in 3D space sequentially at different time frames, which have been acquired by translating a three-axis motorized stage. In 2018, Zhao et al.^42^ used the computer-generated holography based point-spread-function (PSF) technique for wavefront shaping where the axial scanning of the focus was realized digitally using a digital micromirror device. In 2019, Tran et al.^43^ proposed a technique to implement feedback-based wavefront shaping with optical memory effect, where they have shown a lateral distance of 200 ${\upmu }\text {m}$ between focus spots in 3D. However, it is well known that the optical memory effect is limited in its angular range and tilt direction^43,44^. In 2022, Lee et al.^45^ proposed a gradient descent algorithm based 3D color holography in the open air by projecting multiple independent holograms using temporal multiplexing technique. In our work, we have demonstrated *simultaneous* multiple complex hetero-structures formation through tissue like scattering media in 3D volume using a single binary phase mask optimized with R-squared fitness function.

On the experimental side, demonstrations have been conducted using either a nematic liquid crystal SLM (NLC-SLM)^46–49^ or a digital micro-mirror device (DMD)^27,51^. Despite the introduction of different types of algorithms, advanced hardware such as fast cameras, high-resolution NLC-SLMs, or digital micro-mirror devices (DMDs) are still out of reach for most of the research groups due to their high cost. The DMDs have a faster refresh rate ($\sim 23\, \textrm{kHz}$)^46,47^ and low latency. On the other hand, NLC-SLMs have high latency and low frame rate ($\sim 60\, \textrm{Hz}$)^27,51^. However, DMDs can only achieve binary amplitude modulation, which reduces the enhancement factor compared to the phase modulation achieved by either binary FLC-SLMs (two discrete phase levels) or NLC-SLMs (256 discrete phase levels)^51^. The theoretical enhancement factor ($\eta$) of binary phase modulation with FLC-SLM is double compared to DMD^15,51^. Furthermore, the alignment of DMD is difficult due to its sensitivity to oblique reflection, and it is limited to low-intensity pulsed lasers only^48,49^. For an NLC-SLM, phase calibration is mandatory, whereas an FLC-SLM does not require any phase calibration. FLC-SLM is faster than NLC-SLM since it operates in binary mode. FLC-SLM offers a cost-effective alternative with rapid binary phase modulation (up to 4.5 $\textrm{kHz}$), and exhibits increased enhancement compared to DMDs. The use of FLC-SLM for focusing light in scattering media has been shown using the DOPC-based wavefront shaping technique^51^. However, DOPC techniques have some unavoidable drawbacks, such as camera pixels and SLM pixels must be in a near-perfect match which makes alignments far more challenging^23,52^. Furthermore, the most challenging task is that the SLM and camera have to be at the exact mirror conjugate plane^23,52^.

In this article, a cost-effective iterative wavefront shaping system has been designed using binary phase capabilities of FLC-SLM and dual cameras to construct multiple non-similar complex structures at different depths simultaneously in 3D volume using a single binary phase-mask. To achieve multiple complex structures simultaneously, we have utilized the advantages of the R-squared metric as a fitness function in the genetic algorithm. The FLC-SLM has a pixel switching response time of 40 ${\upmu }\text {s}$ with a refresh rate of up to 4.5 $\textrm{kHz}$ at present^53^. The high refresh rate, high-speed pixel switching time, and binary phase features of FLC-SLM can be utilized to advance the resolution in lesser time. We have validated the prototype system using a 120 grit ground glass (GG) diffuser along with 307 ${\upmu }\text {m}$ and 812 ${\upmu }\text {m}$ thick fresh ex-vivo chicken tissues. Multiple complex light structures and gradient contrast light formation with R-squared fitness function will find new applications in 3D holographic display^40,43^, photo-thermal imaging and therapy, fluorescence imaging^7,11^, light sheet microscopy^54,55^, photoacoustic microscopy^56^, and structure illumination microscopy^10^.

## Results and discussion

**Figure 1:**
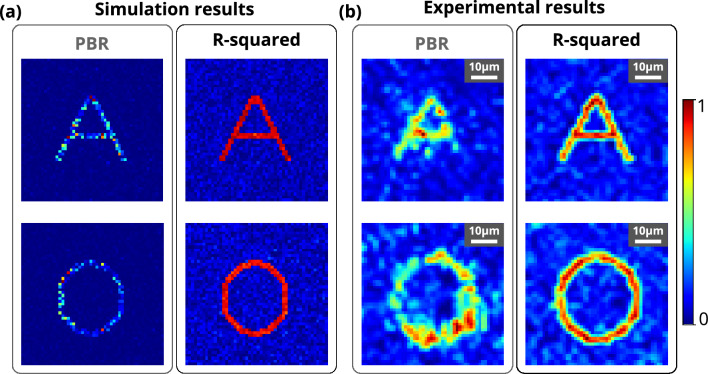
Reconstructed light structures using R-squared and PBR fitness functions. (**a**) Simulation results for reconstructed structures A and O using PBR (left column) and R-squared (right column) fitness functions. (**b**) Experimental results for reconstructed structures A and O using PBR (left column) and R-squared (left column) fitness functions. All the experimental results have a scale bar of 10 ${\upmu }\text {m}$.

**Figure 2:**
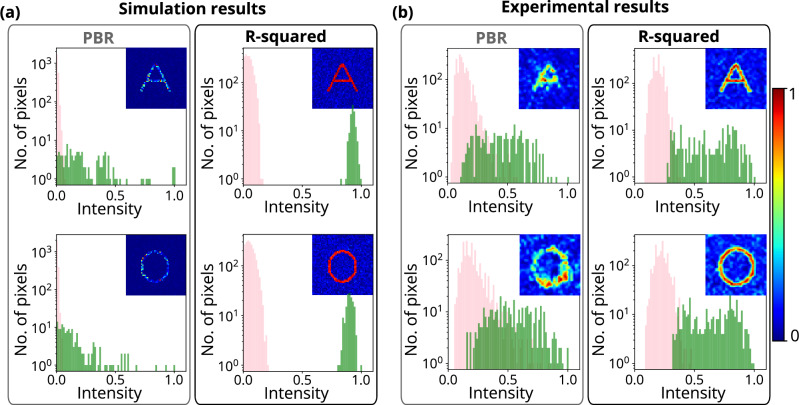
Histogram comparison between R-squared and PBR fitness functions. Simulation and experimental results show reconstructed light structures A and O using PBR and R-squared fitness function and their intensity histogram. The green bars and pink bars represent the target pixels and background pixels, respectively. The histograms clearly show that the intensity of target pixels (green bars) and background pixels (pink bars) has been overlapped and distributed over larger intensity values span while using the PBR fitness function. However, while using the R-squared fitness function, the intensity of target pixels (green bars) and background pixels (pink bars) has been separated or resolved clearly with a narrower intensity distribution.

The fitness function in iterative optimization algorithms is essential to reach the optimum solution. It has been observed that the data regression assisted R-squared fitness function, and the most commonly used peak-to-background ratio (PBR) fitness function perform differently based on the complexity of the structure at the region of interest (ROI). Figure 1 shows that the PBR-based fitness function has not been able to resolve the structure and did not achieve uniform intensity at the target pixels for complex structures such as alphabet letters **A** and **O**. A uniform intensity distribution over all pixels at the target location is essential to resolve complex structures. In the experiment, the PBR-based fitness function is not able to construct a complex structure such as the alphabet letter **A** and **O** (see Fig. 2). However, the R-squared metric-based fitness function outperforms PBR in terms of constructing the structures by enhancing the resolution and structural uniformity. The insets of Figs. 1 and 2 show the images of the constructed structures **A**, **O** and histograms of the intensity distribution in the target area for both the R-squared metric and the PBR fitness functions, respectively. The R-squared metric is a measure of variance between two data sets^57,58^, and it has been used frequently in machine learning^59^.

### R-Squared metric and regression model analysis

Regression analysis is a statistical method that estimates the relationship between dependent (reference image (*I*)) and independent (obtained image (*S*)) variables^58^, where $S =\left\{ S_j \mid j=1,2, \ldots n\right\}$ represents the intensity of pixels of the obtained image and $I =\left\{ I_j \mid j=1,2, \ldots n\right\}$ represents the intensity of pixels of the reference image (*I*). A non-linear regression model $f =\left\{ f_j \mid j=1,2, \ldots n\right\}$ is formulated with the pixels of the obtained camera image and the pixels of the reference image as;

1 $$\begin{aligned} f_j = b_0 + {b_1}{S_j} + {b_2}{S_j}^2 + {b_3}{S_j}^3+\cdots +\cdots \end{aligned}$$

where $b_0$, $b_1$ represent the linear parameters and $b_2$, $b_3$ represent the higher order parameters in the regression model. However, for simplicity, we have restricted it within the linear regression. The linear regression model is expressed as follows;

2 $$\begin{aligned} f=\left[ \begin{array}{c} {f}_1 \\ {f}_2 \\ \vdots \\ {f}_n \end{array}\right] =\left[ \begin{array}{c} b_0+b_1 S_1 \\ b_0+b_1 S_2 \\ \vdots \\ b_0+b_1 S_n \end{array}\right] =\left[ \begin{array}{cc} 1 &{} S_1 \\ 1 &{} S_2 \\ \vdots &{} \vdots \\ 1 &{} S_n \end{array}\right] \left[ \begin{array}{l} b_0 \\ b_1 \end{array}\right] \end{aligned}$$

**Figure 3:**
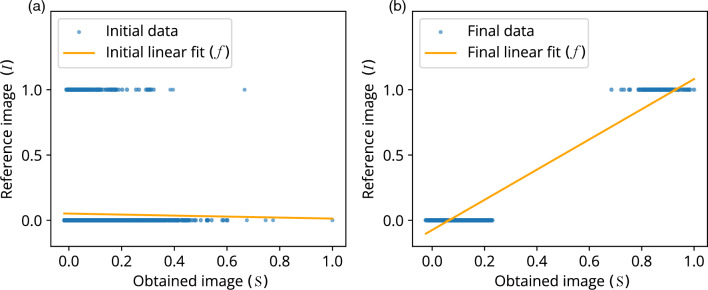
Progression of R-squared linear fit in the simulation after 700 generations. (**a**) Linear fit (*f*) of obtained image (*S*) vs. reference image (*I*) before optimization, which clearly shows a poor linear fit (orange line). (**b**) Linear fit (*f*) of the obtained image (*S*) vs. reference image (*I*) after optimization. For reference image (*I*) on Y-axis, the intensity values 0 and 1 represent the background and target pixel intensities, respectively.

Figure 3 shows the linear regression for the R-squared fitness function. Figure 3a represents the initial linear fit of the reference image (*I*) and obtained image (*S*) before starting optimization. Whereas, Fig. 3b shows the optimized linear fit of the reference image (*I*) and obtained image (*S*) after 700 iterations. Similarly, higher-order regression can also be analyzed further with a suitable non-linear regression model.

Further, the linear regression model described above is used to formulate the R-squared fitness function as follows;

3 $$\begin{aligned} R^2 = 1 - \frac{\sum _{j=1}^\text {N} (I_j - \textit{f}_j)^2}{\sum _{j=1}^\text {N} ({I_j - {\overline{I}}})^2},\qquad \textit{where} \,\,\,{\overline{I}} = \frac{1}{\text {N}} \sum _{j=1}^\text {N} I_j \end{aligned}$$

This R-squared coefficient value lies between 0 and 1. It quantifies the relationship between the movement of a dependent variable and an independent variable. Its coefficient value 1 refers to a perfect match between the two sets of data, and the value close to 0 represents no linear relationship between the two data sets^57^. Detailed analyses of structural uniformity, resolution enhancement, and background noise suppression in the presence of varying noise percentages for the PBR and R-squared fitness function are shown in Figs. S3–S4 (Supplementary material).

### Cross-correlation metric to quantify structural light

A mathematical function or metric is essential to quantify the uniformity of the constructed light structure and its similarity test with respect to a reference image. For this, the cross-power spectrum has been implemented to estimate the cross-correlation metric (CCM) using the obtained image (*S*) and the reference image (*I*).

Cross-power spectrum analysis is a technique commonly used in signal processing to quantify the similarity between two signals or images^60^. It compares the power spectra of two signals or images to identify common frequency components to measure the similarity. The cross-power spectrum is calculated by multiplying the complex conjugate of the Fourier transform *S*(*u*, *v*) of the obtained image *S*(*x*, *y*) by the Fourier transform *I*(*u*, *v*) of the reference image *I*(*x*, *y*). This gives a complex-valued cross-power spectrum, which is further normalized by dividing it by the magnitude of the cross-power spectrum. The normalized cross-power spectrum $C_P(u,v)$ of the obtained image *S*(*x*, *y*) and the reference image *I*(*x*, *y*) is calculated as follows;

4 $$\begin{aligned} C_P(u,v) = \dfrac{ I^{*}(u,v) \cdot S(u,v) }{\left| I^{*}(u,v) \cdot S(u,v) \right| } \end{aligned}$$

Similarly, for normalization, the power spectrum $R_P(u,v)$ of the reference image (*I*) is calculated by multiplying its Fourier transform with its complex conjugate and, then normalizing it as;

5 $$\begin{aligned} R_P(u,v) = \dfrac{ I^{*}(u,v) \cdot I(u,v) }{\left| I^{*}(u,v) \cdot I(u,v) \right| } \end{aligned}$$

The $C_P(u,v)$ and $R_P(u,v)$ are in the frequency domain. To visualize and interpret the results in the spatial domain, the inverse Fourier transform converts the spectra back into the spatial domain $C_P(x,y)$ and $R_P(x,y)$. Finally, a cross-correlation metric (CCM) is calculated by dividing the maximum value of $\left| C_p(x,y) \right| )$  with the maximum value of $\left| R_p(x,y)\right|$. The final cross-correlation metric (CCM) is written as follows;

6 $$\begin{aligned} \text {Cross-correlation metric} = \dfrac{max(\left| C_p(x,y) \right| )}{max(\left| R_p(x,y)\right| )} \end{aligned}$$

**Figure 4:**
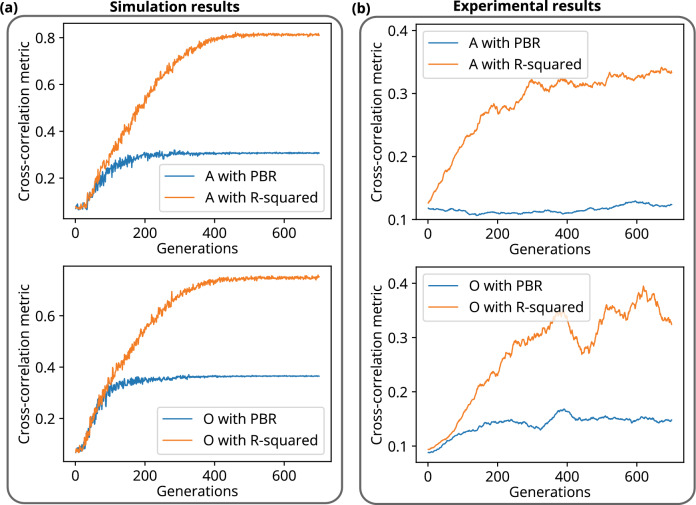
Cross-correlation matric plots for the construction of structures A and O. Evolution of cross-correlation matric with generations for R-squared and PBR fitness functions, (**a**) simulation and (**b**) experiment.

The cross-correlation metric measures the similarity of two images. If the metric variable is close to 1, the images are similar, while if it is close to 0, they are significantly different^61^. Figure 4 shows the analysis of the metric value using simulation and experimental data over 700 generations for both the PBR and R-squared fitness functions.

### Characterization of experimental setup and formation of 2D/3D complex structures through biological tissue media

**Figure 5:**
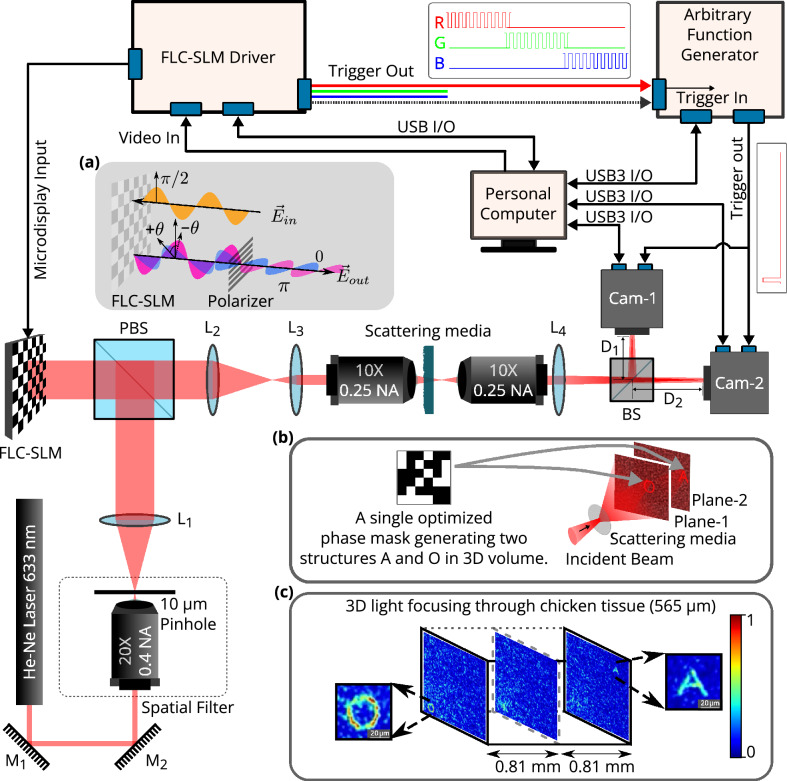
Schematic of the experimental setup. Where, $\mathrm {{M_1}}$ and $\mathrm {{M_2}}$: mirrors 1 and 2. $\mathrm {L_1}$, $\mathrm {L_2}$, $\mathrm {L_3}$ and $\mathrm {L_4}$: lenses 1, 2, 3 and 4. PBS: polarising beamsplitter, BS:50:50 beamsplitter. Cam-1 and Cam-2: Camera-1 and Camera-2, placed at distances $\mathrm {D_{1}}$ and $\mathrm {D_{2}}$ from the 50:50 BS respectively. Furthermore, (**a**) Working principle of the binary phase based FLC-SLM, (**b**) Illustration for multiple complex hetero-structures formation simultaneously in 3D space using a single optimized phase mask, (**c**) Experimental results for 3D complex hetero-structure formation through chicken tissue with an optimized single phase-mask.

The detailed schematic of the experimental system-design with various hardware building blocks, tissue samples for the experiment, and the constructed 3D volume image is shown in Fig. 5. The system design consists of a master controller, i.e., the FLC-SLM hardware driver. This hardware driver is connected further with the responders, i.e., the FLC-SLM’s microdisplay unit and the arbitrary function generator, which triggers both the cameras. The light from a He–Ne laser of wavelength 633 nm passes through a spatial filter and falls on the SLM. Subsequently, the wavefront modulated by the FLC-SLM propagates through a series of optical components and falls on the scattering media. To facilitate the formation of multiple complex hetero-structures *simultaneously* at different depths in the 3D volume, a beam splitter is used to split the speckle field into two parts. These two parts are imaged by cameras placed at two different depths. Camera-1 is placed at distance $\mathrm {D_1}$, which has the option of moving back and forth. Camera-2 is placed at distance $\mathrm {D_2}$ to visualize the 3D volume. Furthermore, a set of sequential hardware operation instructions are sent from the personal computer to the FLC-SLM display head and the cameras for acquiring the output speckle field generated by the tissue sample. The working principle of the FLC-SLM is shown briefly in Fig. 5a and a more detailed overview is shown in Fig. S14 (Supplementary material).

The experiment has been performed with the developed system, where both the PBR and the R-squared fitness function have been tested to focus the complex 2D as well as 3D structures. A commercial 120-grit GG diffuser has been used as the scattering medium. Chicken tissue samples of thickness 307 ${\upmu }\text {m}$ and 812 ${\upmu }\text {m}$ have been used for demonstration.

**Figure 6:**
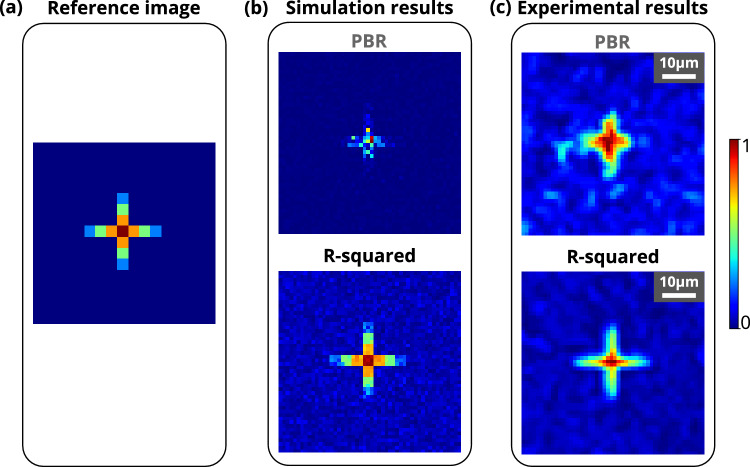
Formation of the ‘plus’ sign structure consisting of gradient grayscale values. (**a**) Reference ‘plus’ sign structure image consisting of four different grayscale based target pixels and one grayscale level based background pixel. (**b**) Simulation results for the ‘plus’ sign structure reconstructed using PBR and R-squared fitness functions. (**c**) Experimental results for the ‘plus’ sign structure reconstructed using PBR and R-squared fitness functions.

Formation of the complex patterns through the GG diffuser is shown in Fig. 1. It has been observed that standard PBR is not able to focus complex structures such as the alphabet letters **A** and **O** clearly, while the R-squared fitness function is able to form well-resolved **A** and **O** through a highly scattering 120-grit GG diffuser. The word ‘IISER’ has also been constructed through the 120-grit GG diffuser to demonstrate a more complex 2D structure formation. The result is shown in Fig. S1 (Supplementary material), where it has been observed that the R-squared fitness function efficiently forms the structure IISER, while PBR is not able to form it. Furthermore, a plus sign structure consisting four gradient grayscale based target pixels along each arm and one grayscale based background pixels has also been constructed through 120-grit GG diffuser. The results for plus structure are shown in Fig. 6, where it has been observed that the R-squared fitness function is able to construct gradient contrast along each arm of plus sign structure in both simulation and experiment, while PBR is not able to form it. It has also been observed that the lower value contrast, which is near the background intensity, spreads all around the structure while PBR fitness function is considered.

**Figure 7:**
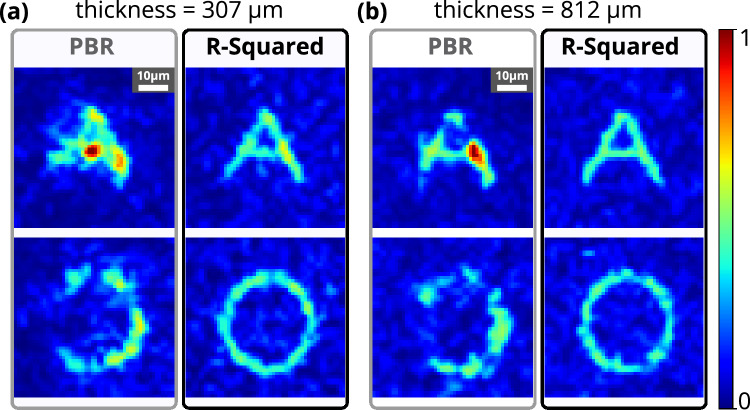
Experimental results for complex structure formation in 2D space using chicken tissues of different thicknesses. Comparison between reconstructed alphabet structures **A** and **O** using the PBR and R-squared fitness functions with chicken tissue of thickness 307 ${\upmu }\text {m}$ and 812 ${\upmu }\text {m}$. All images in this figure have the same scale bar (10 ${\upmu }\text {m}$).

The stability of the experimental setup has been demonstrated with chicken tissue samples of thickness 307 ${\upmu }\text {m}$ and 812 ${\upmu }\text {m}$. Figure 7 shows the formation of complex structures through chicken tissues for the standard PBR and the R-squared fitness function. Similar to the GG diffuser results, the R-squared fitness function outperforms the standard PBR in terms of advancing the resolution, structural uniformity, and background suppression for complex structure formation through chicken tissue. As the thickness of the tissue sample has been increased to 815 ${\upmu }\text {m}$, still R-squared fitness function has shown well-resolved structure formation compared to the standard PBR.

### Simultaneous formation of multiple complex hetero-structures in 3D space through tissue

**Figure 8:**
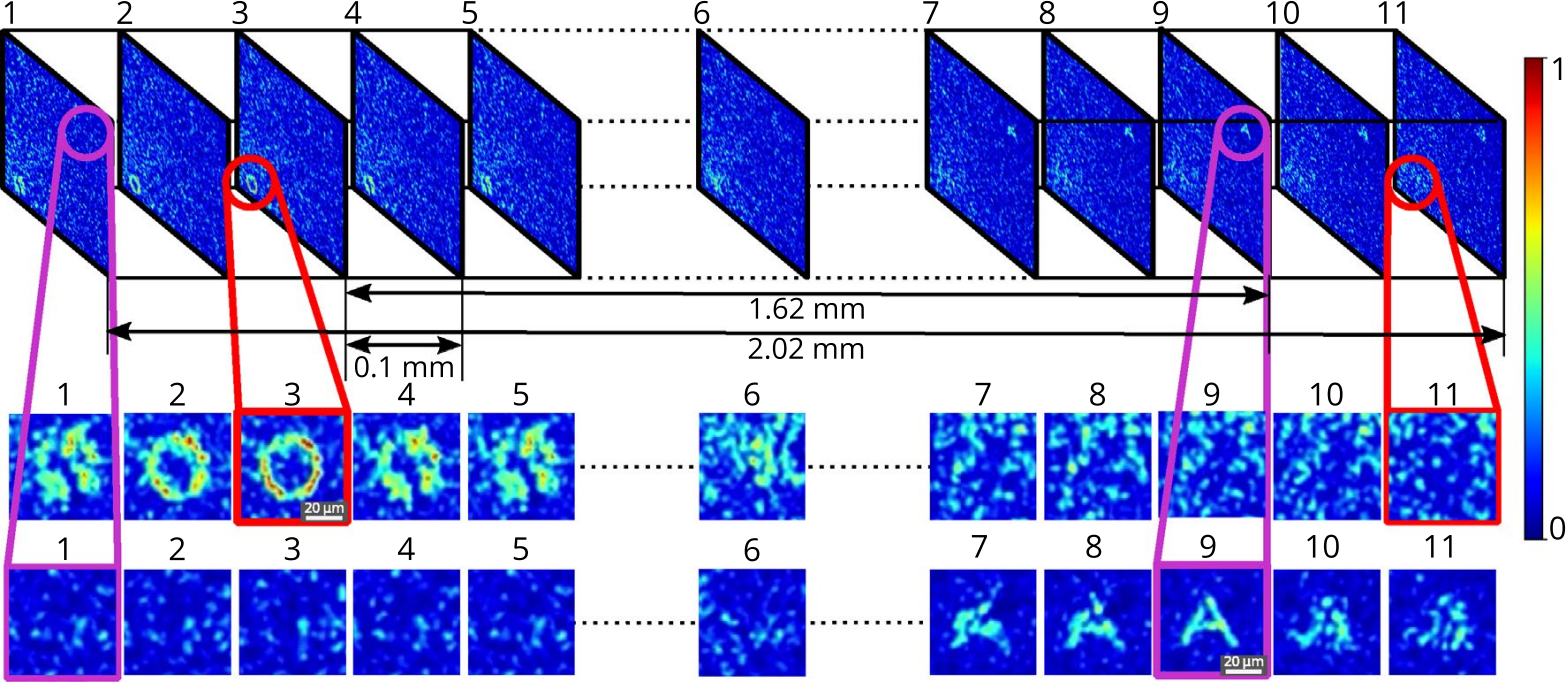
Experimental results for the simultaneous formation of multiple hetero-structures in 3D space. Figure shows the slices of 3D volume for constructed complex structures through chicken tissue, where alphabets **A** and **O** structures are formed in different planes. The axial distance between highly resolved structures **O** ($\mathrm {3{rd}}$ plane) and **A** ($\mathrm {9{th}}$ plane) is 1.62 $\text {mm}$, and the lateral distance is 266 ${\upmu }\text {m}$.

In this work, an experimental system with dual cameras has been proposed (Figs. 5, 10), which simultaneously facilitates the construction of multiple complex structures in a 3D space (Fig. 8). The proposed setup is able to construct structures in a much larger 3D volume compared to the volume covered with angular range and tilt direction of the optical memory effect. With the R-squared fitness function and the experimental setup, multiple complex structures have been constructed *simultaneously* at different planes of 3D volume by displaying an optimized single phase-mask on the FLC-SLM. A fresh chicken tissue of thickness 565 ${\upmu }\text {m}$ has been used as a scattering medium. A phase mask has been optimized using the R-squared fitness function in the genetic algorithm^35^ and displayed on the FLC-SLM to form complex structures in multiple planes in 3D space. Figure 8 shows the 3D volume slice images of the formation of A and O structures through chicken tissue using a single optimized phase mask. The axial and lateral distances between the two complex objects have been kept at 1.62 $\text {mm}$ and 266 ${\upmu }\text {m}$, respectively. The resolution of the system has been estimated with the objective lens (10$\times$, 0.25 NA) at wavelength 633 nm and found to be $88.15\,\pm 2\,{\upmu }\text {m}$. The detailed system resolution and more complex volume imaging with multiple images in 3D volume are shown in the Supporting information (Section S4.3, Fig. S16).

## Conclusion and perspectives

In the field of wavefront shaping, it is crucial to pursue objectives such as increasing the efficiency of light transmission through turbid media, enhancing resolution, developing structural light uniformly, forming gradient contrast in light structures, and achieving a precise focus that allows 2D or 3D light structure formation through scattering media. Our experimental setup demonstrates notable advancements in resolution and structural uniformity, which facilitates the formation of multiple light structures within a 3D volume through the scattering media (Fig. 8).

**Figure 9:**
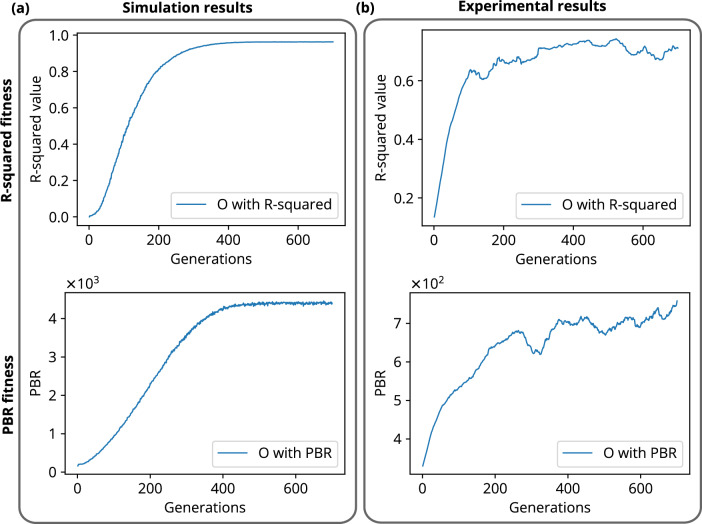
R-squared and PBR fitness plots for the construction of structures O. (**a**) Simulation results for the progression of fitness values over generations for both R-squared and PBR fitness functions, (**b**) experimental results for the progression of fitness values over generations for both R-squared and PBR fitness functions.

A data regression model based R-squared fitness function has been introduced into the algorithm and implemented in the FLC-SLM based iterative binary phase modulation system (Fig. 3). The prototype system with R-Squared fitness function has shown remarkable performance in improving structural uniformity and resolution (Figs. 1, 2, 6, 7,  8). The developed cost-effective and calibration-free (wavelength independent phase calibration) iterative wavefront shaping system along with R-squared fitness function has been validated with a 120-grit GG diffuser along with fresh ex-vivo chicken tissue samples of thickness 307 ${\upmu }\text {m}$ and 812 ${\upmu }\text {m}$ (Figs. 7, 8). A cross-correlation based metric (CCM) has also been analyzed to quantify the structural similarity of the constructed light structures. Simulation results show that R-squared fitness function has achieved up to 81.4% CCM value where PBR has achieved below 30.1% CCM value for light structure **A** (Fig. 4). Similarly, experimental results show that R-squared has been able to achieve up to 33% CCM value whereas PBR has achieved only 11% for light structure **A** (Fig. 4). The standard deviation analysis shows that R-squared fitness function has achieved substantial improvement in the uniformity of intensity at target and background pixels compared to the PBR fitness function (Figs. S10, S11, Supplementary material). The fitness value for PBR has shown progression with the generation, but it failed to construct the structure, whereas R-squared fitness function has shown well-resolved structure (Fig. 9). The proposed method has demonstrated robust noise tolerance while varying the noise percentage from 10 to 100%, and the results are shown in Figs. S2, S3 (Supplementary material). The impact of  input modes (*N*) variation has also been  analyzed for the PBR and R-squared fitness functions in both the simulation and experiment. Detailed results for the input modes analysis are shown in Figs. S4–S9. The designed system with the dual cameras and R-squared fitness function has constructed high-resolution, non-similar multiple complex structures (A/O shapes) simultaneously at different depths in 3D using an optimized single phase-mask. This work may find potential applications in 3D confocal microscopy, 3D photoacoustic microscopy, photolithography, structured light illumination microscopy, 3D holography, and photothermal therapy. However, the efficiency of the SLM decreases with increasing the number of complex structures as a result of the limited availability of optimized input modes.

The other advanced functionalities of the FLC-SLM, including RGB data transfer using three color channels and its wavelength-independent phase calibration, provide an advantage in designing new experiments. The fast pixel-switching time ($40 \, {\upmu }\text {s}$) and a high refresh rate of $4.5 \, \textrm{kHz}$^53^ make the FLC-SLM suitable for applications such as tissue imaging, live cell imaging, and photoacoustic microscopy. Despite advancements in algorithms and SLM refresh rate, the operating speed of the entire system is bottlenecked by the slow data transfer rate between the camera and the PC. However, the delay in data transfer from the camera to the PC can be reduced drastically using a multichannel data transfer protocol like *CoaXPress*. A faster acquisition speed will further reduce the number of iterations required to reach convergence, as it will reduce the noises generated due to beam shifts, temperature fluctuations, and the camera sensor’s response. These advantages and cost-effectiveness make the system more suitable for designing various complex wavefront shaping experiments.

## Methods

### Computational model

The output complex field $E(d^{'})$ obtained through a scattering media of transmission function $T(d,d^{'})$, can be written as $E(d^{'})$=$\sum _{d}T(d,d^{'}) E(d)$. Where the incoming light field is denoted by *E*(*d*). A transmission matrix (*T*) of dimensions $M \times N$ models wavefront scattering through disordered media. Here, *T* is generated by a complex Gaussian random matrix. The equation for the calculation of the output modes *M* can be written as^16,25^;

7 $$\begin{aligned} E_m = \sum _{n}^{N} t_{mn} A_n e^{i{\phi }_n} \end{aligned}$$

where $A_n$ and ${\phi }_n$ are the amplitude and phase of an input mode (*n*), respectively, and $t_{mn}$ is a particular element of the transmission matrix *T*. The amplitude of the complex field is chosen as $A_n=1/\sqrt{N}$. Therefore, the intensity ($I_m$) at a particular output mode at the camera with added noise can be written as;

8 $$\begin{aligned} I_m = \frac{1}{N}\Bigg |{ \sum _{n}^{N} t_{mn} e^{i{\phi }_n}}\Bigg |^2 +\, \delta \quad \text {where,} \quad \delta = \frac{\Gamma \%}{100} \times {\mathscr {N}}(\mu _T,\,\sigma _T)\, <I_o> \end{aligned}$$

Here, a noise ($\delta$) is added to mimic the experimental environment. $\Gamma$ represents the percentage of added noise with respect to the initial average intensity $<I_o>$. ${\mathscr {N}}(\mu _T,\sigma _T)$ represents a random number generated from a normal distribution with mean ($\mu _T$) and standard deviation ($\sigma _T$). The parameters for GA are set according to the optimized values described in the literature^15,37^. In feedback algorithms, especially for GA, intensity based fitness function is considered as ${I^{T}_{i}}/ {I^{{\bar{B}}}_{initial}}$, where $I^{T}_{i}$ is the intensity at target pixels for *i*th generation and ${I^{{\bar{B}}}_{initial}}$ is the initial average intensity at the background pixels^16,32^. Whereas, PBR based fitness function is considered as ${I^{T}_{i}}/ {I^{{\bar{B}}}_{i}}$, where $I^{T}_{i}$ is the intensity at target pixels for *i*th generation and $I^{{\bar{B}}}_{i}$ is the average intensity at the background pixels for *i*th generation^31,37^. However, this article has considered the R-squared metric as a fitness function, which has been discussed thoroughly in the first part of the results and discussion section.

The simulation model has been designed in the Python 3 programming language and NumPy has been used to process the matrices. As per the input mode analysis, an optimized matrix of dimensions $250\times 250$ has been considered as the input modes matrix that corresponds to total $N=62,500$ input modes in the simulation (Figs. S4–S7). On the output side, a matrix of dimensions $50\! \times \!50$ has been considered as the output mode matrix that provides $M=2500$ output modes ($\vec {E}_{out}$). The transmission matrix (*T*) of dimensions $M\times N$ has been generated using a complex Gaussian random distribution ($\mu _T = 0$ and $\sigma _T = 0.1$) to mimic light scattering. In addition, a 30% noise $\delta$ has been added to the output mode intensity to simulate the experimental conditions. In the experiment, an optimized input modes matrix of dimensions $320\times 256$ has been considered (Figs. S8, S9). Furthermore, a matrix of dimensions $50\! \times \!50$ has been considered as the output mode matrix in the experiment.

In the beginning of the algorithm, a population (*P*) of random binary phase masks has been generated using a discrete uniform distribution of values 0 and 255, which correspond to the 0 and $\pi$ phase, respectively. A population size of 200 has been chosen as it provides a good trade-off between speed and enhancement. Two parents $\vec {P}_i$ and $\vec {P}_j$ have been selected with a biased probability toward a higher fitness value. The descending order of the phase masks has been ranked according to their fitness value, which was later used for the selection of parents. The crossover rate ($r_c$) has been kept at the standard value of 50%. The initial mutation rate has been fixed at 1%, which decays exponentially with a constant decay rate ($\lambda$)^15,37^.

### Experimental system design with FLC-SLM

**Figure 10:**
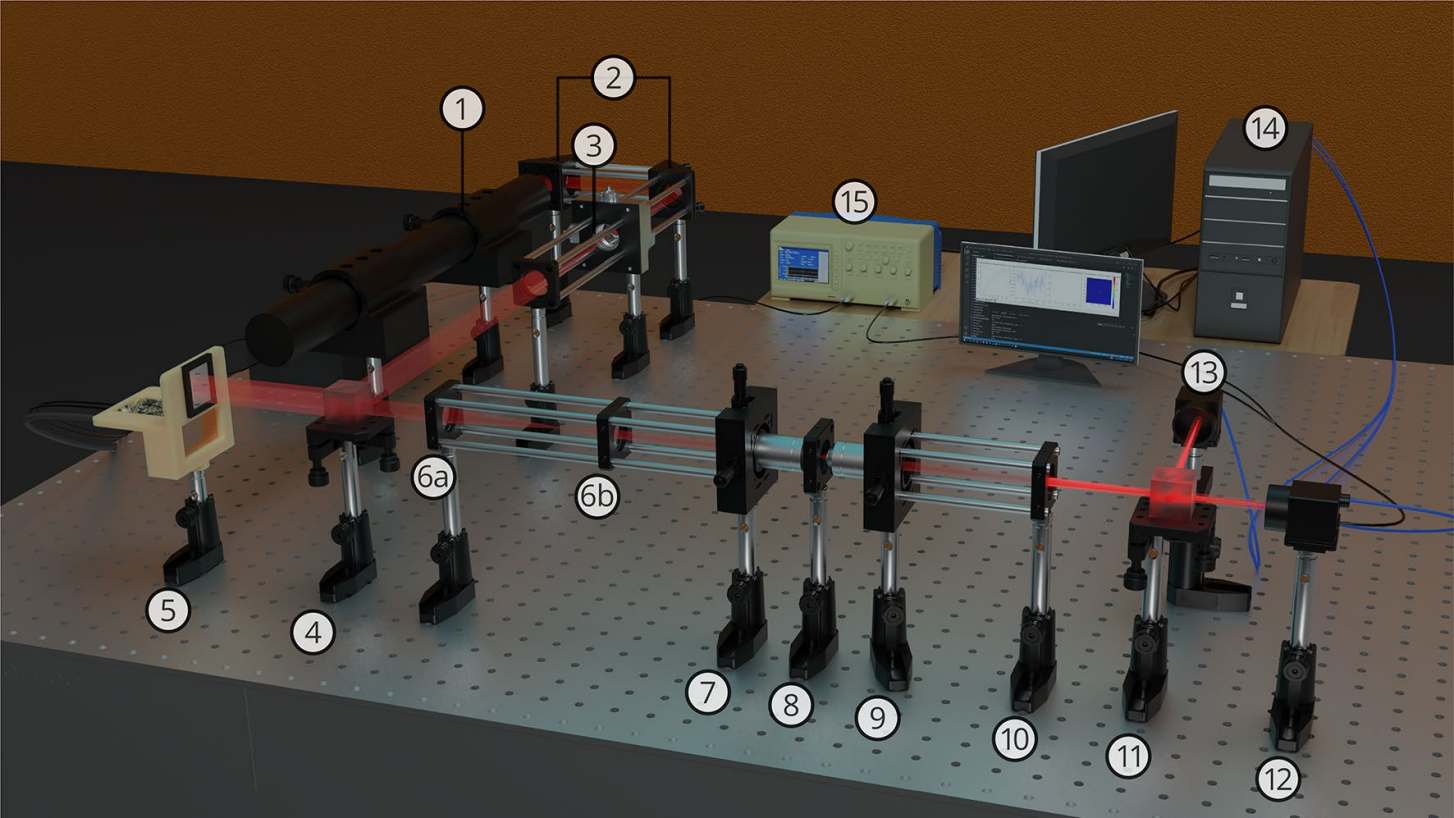
3D schematic of experimental setup. Components: 1. He–Ne laser, 2. Mirrors $\mathrm {M_1}$ and $\mathrm {M_2}$, 3. Spatial filter, 4. Polarizing beam splitter (PBS), 5. FLC-SLM, 6a–6b. 4F setup, 7. 1st objective, 8. Scattering media, 9. 2nd objective, 10. Lens ($f = 50\, \text {mm}$), 11. 50:50 Beam splitter, 12. CMOS camera-1, 13. CMOS camera-2, 14. PC, and 15. Arbitrary function generator.

A detailed schematic of experimental setup is shown in Figs. 5 and 10. A 12 mW He–Ne laser (633 nm, Newport), consisting of vertical linearly polarized light with a polarization ratio of 500:1, is used in the built system. The alignment of the laser beam has been done with the help of two flat mirrors $\mathrm {M_1}$ and $\mathrm {M_2}$. Along the path, a spatial filter system (Thorlabs, KT311/M) is placed consisting of a pinhole ($\phi =10\, {\upmu }\text {m})$ and objective (20$\times$, Numerical Aperture (NA) = 0.40) for eliminating the higher-order noise from the beam. Thereafter, the spatially filtered diverging beam is collimated by a lens $\mathrm {L_1}$ $(f = 250 \, \text {mm})$ to get a pure flat beam profile on the surface of FLC-SLM. A polarising beam splitter (PBS) and FLC-SLM (ForthDD, SXGA-R5) are used for the wavefront modulation. The modulated wavefront is passed through a 4*F* setup and enters into an objective (10$\times$, $\text {NA} = 0.25$) which transmits the wavefront through the scattering media. The power of the incident beam before entering into the tissue sample has been measured and found to be 0.74 mW. A second objective (10$\times$, $\text {NA} = 0.25$) is placed behind the scattering media. For the simultaneous construction of multiple complex structures in 3D volume, the CMOS camera-1 (Thorlabs, DCC3260C) and CMOS camera-2 (Basler acA800-510uc) are placed at distance $\mathrm {D_1}$ and $\mathrm {D_2}$, respectively, to acquire images at different depths in 3D space and make the feedback signals for the algorithm. The CMOS camera-1 has the option to move back and forth to construct multiple complex structures at more than two different depths.

The signal from the PC to the SLM driver module is sent via a video card. Each image is a combination of 24 bit-planes, i.e., 24-bit information per pixel and 8-bit per channel (RGB). The hardware module of the SLM splits the RGB signal into 24 single-bit black-and-white images. These 24 single-bit images are sent and displayed on the SLM screen sequentially. In conclusion, a total of $24\times 60 = 1440$ binary images are displayed on the SLM screen in 1s with these setting parameters. Each bit plane is displayed on the SLM screen for a duration of 219.02 ${\upmu }\text {s}$. The hardware driver module of the SLM is programmed to generate an output electrical signal which becomes high or low in synchronization with the display of each bit plane. This signal is passed to the function generator to generate a new signal with $+3\,\textrm{V}$ to trigger the two CMOS cameras. The other advanced features of the FLC-SLM, such as three color channels, can be utilized either collectively or individually. The two cameras have been triggered using the function generator to construct multiple simultaneous complex structures in 3D space.

### Preparation of chicken tissue samples for the experiment

The experiment was conducted without the use of live animals. A part of fresh skinless chicken (weight = 2.62 $\text {kg}$, age = 10 weeks, measured density = 0.92 $\mathrm {g/cm}^3$) was procured from the local market. The chicken thigh was kept in the freezer for 4 hours at a constant temperature of $-14 \, ^{\circ }\textrm{C}$ to facilitate the slicing. A sterilized surgical scalpel was used to section the chicken tissue into multiple slices. The measured thickness of the sliced chicken tissues has been found to be 307 and 812 ${\upmu }\text {m}$ for 2D structure formation, and 565 ${\upmu }\text {m}$ for 3D multiple structures formation. The sliced chicken muscle was sandwiched between two microscope glass cover-slips. A drop of Glycerol was used to preserve the sample and prevent it from dehydration.

### Supplementary Information

Supplementary Information.

## Data Availability

The original contributions presented in the study are included in the article and the Supplementary Material; further inquiries can be directed to the corresponding author.
