# Supplementary material for: Formation of multiple complex light structures simultaneously in 3D volume using a single binary phase mask
Source: Sci Rep. 2023 Oct 7;13:16951. doi: 10.1038/s41598-023-42087-0 (PMC10560216; doi:10.1038/s41598-023-42087-0)
Supplement: Supplementary file 1 — Supplementary Information. [file 41598_2023_42087_MOESM1_ESM.pdf]

Supplementary material : Formation of multiple complex light structures simultaneously in 3D volume using a single binary phase mask

Amit Kumar, Sarvesh Thakur, and S. K. Biswas*

Bio-NanoPhotonics Laboratory, Department of Physical Sciences, Indian Institute of Science Education and Research Mohali, Knowledge City, Sector 81, SAS Nagar, Manauli PO 140306, India
*skbiswas@iisermohali.ac.in

S1 Reconstruction of word 'IISER' together in the experiment using a single binary phase mask

The word 'IISER' has been constructed through the 120-grit GG diffuser to demonstrate a more complex 2D structure formation. It has been observed that the R-squared fitness function is able to form the structure IISER, while PBR fitness function is not able to form the structure.

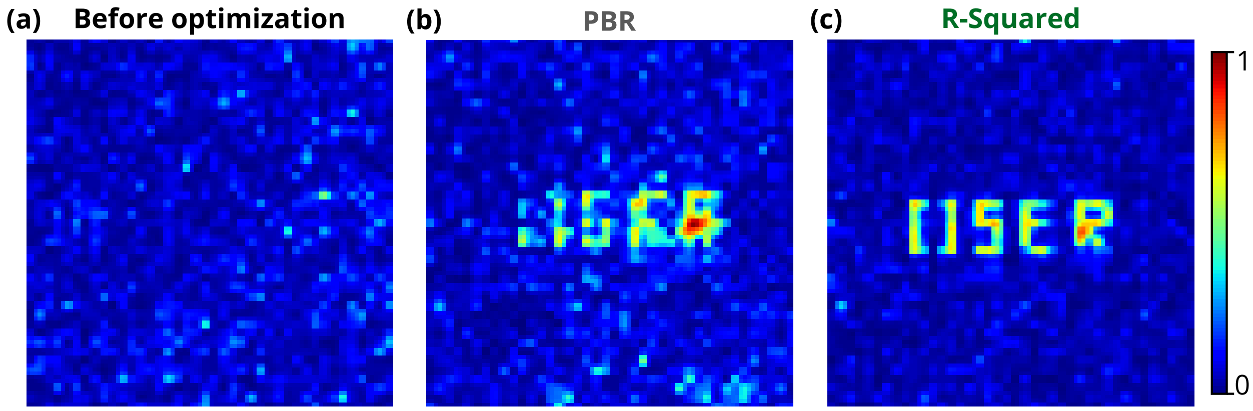

Figure S1: Reconstruction of a couple of complex light structures using PBR and R-squared fitness function. The word 'IISER' has been constructed through 120 grit ground glass diffuser.v **(a).** shows the speckle image before optimization. **(b).** shows the several alphabet letters (IISER) reconstructed together using the PBR fitness function, and **(c).** shows the several alphabet letters (IISER) reconstructed together using the data regression assisted R-squared fitness function.

S2 Simulation details

S2.1 Noise comparison in simulation

To keep the noise a physical quantity, the intensity values were clipped at zero. The algorithm was tested in the presence of high amount of noise. Noise was calculated with respect to the initial average intensity $< I_o >$ at output modes. The output mode with noise percentage $\Gamma\%$ is given as;

$$I_{out} = I_{\substack{without \\ noise}} + \frac{\Gamma\%}{100} \times \mathcal{N}(\mu_T, \sigma_T) < I_o > \tag{S1}$$

Where $I_{\substack{without \\ noise}}$ is intensity at output mode before adding noise. I_{out} is intensity at output mode after adding the noise percentage $N\%$. $\mathcal{N}(\mu_T, \sigma_T)$ is Gaussian noise with $\mu_T = 0$ and $\sigma_T = 1/3$.

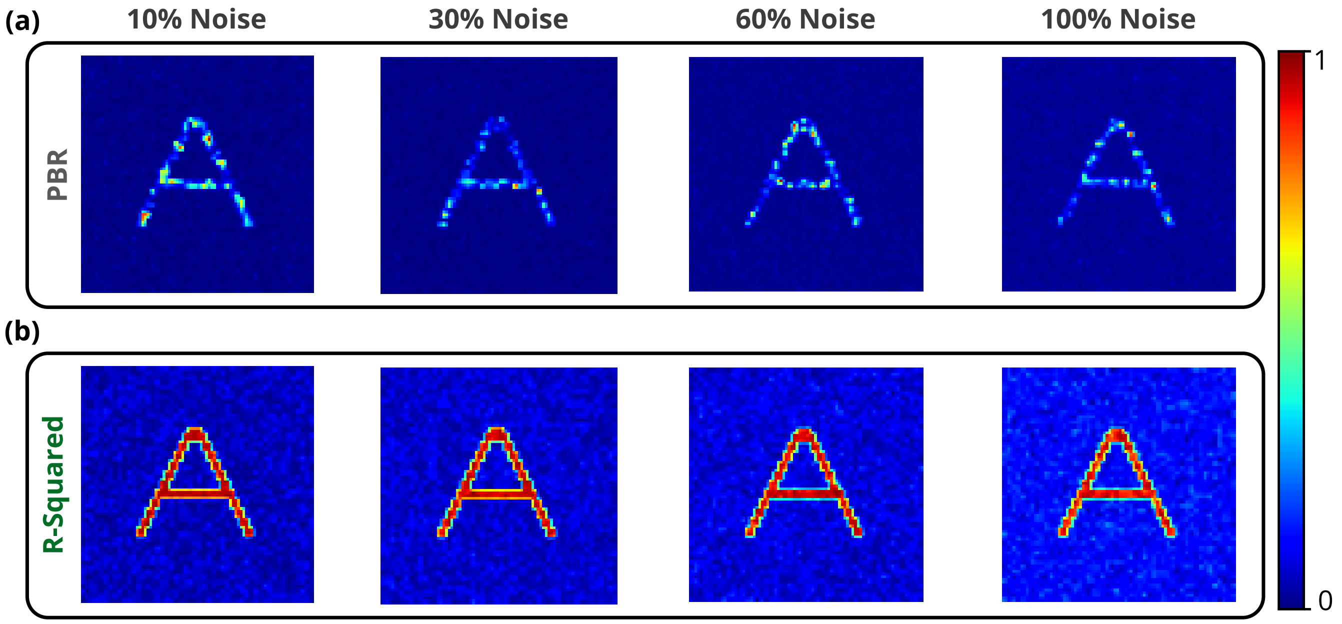

Figure S2: Study of noise in simulation model data and its effect on complex structure formation. Figure shows the images for the structural uniformity and resolution comparison in the presence of 10%, 30%, 60% and 100% noise with respect to the initial average intensity for the PBR and R-squared fitness function.

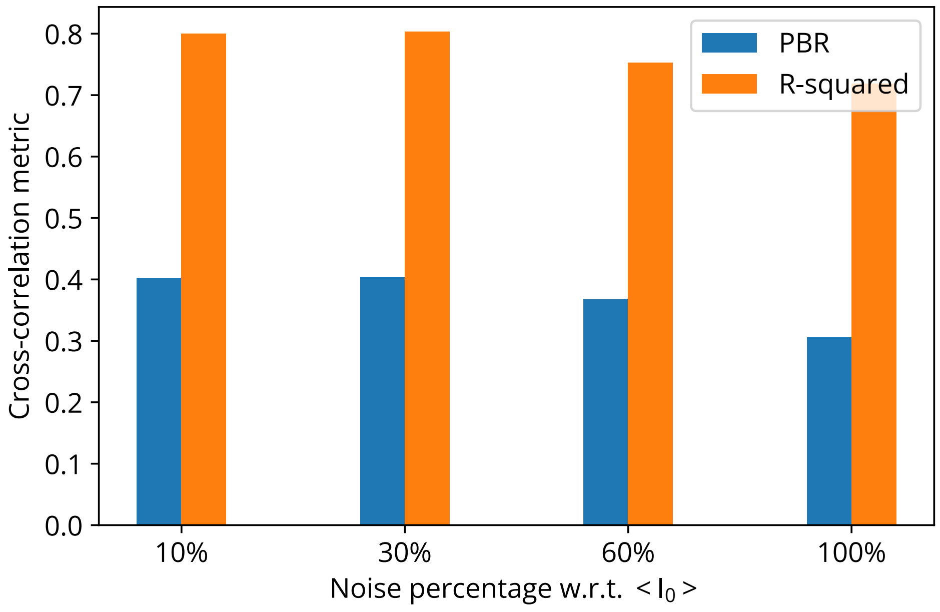

Figure S3: The performance of the PBR and R-squared fitness functions in simulation model with increasing noise percentage. The simulation model was analysed for R-squared and PBR fitness functions using cross-correlation metric with different noise percentages 30%, 60%, 60%, and 100% with respect to the initial average intensity.

S2.2 Comparison of input modes

The effect of input modes is analyzed on the PBR and R-squared fitness functions while increasing the size of the input mode matrix (N). The input mode matrix (N) is a squared matrix of dimensions $\sqrt{N} \times \sqrt{N}$. The simulation results are described in Figs. S4, S5, S6, and S7. Here, Fig. S4 shows the images of the reconstructed structure A using the PBR and the R-squared fitness function while increasing the input modes from 25×25 to 250×250 in simulation. Fig. S5 shows the effect on the cross-correlation metric while increasing the input modes using the PBR and R-squared fitness function in simulation. Fig. S6 shows the change in the R-squared fitness value with increasing input modes, where the theoretical maximum fitness is also shown with the orange line. Fig. S7 shows the change in the PBR fitness value as the input modes increase in simulation. Furthermore, the change in the theoretical enhancement factor (η) is also shown through the dashed orange curve as the input modes increase. Simulation results also show that when the input modes increase from 25×25 to 100×100, the fitness value of the PBR appears to follow the η-curve. If the input modes are further increased above 100×100, the fitness value of the PBR does not increase according to the

η-curve after this and begins to saturate further, and this behavior has been reported by Anderson *et al.* in 2014[1]. In genetic algorithms, most of the previous literature has explored the input modes from 16×16 to 64×64[2–8]. The experimental results are described in Figs. S8 and S9. Fig. S8 shows the effect on the cross-correlation metric and Fig. S9 shows the effect on the R-squared fitness value while increasing the input modes from 40×32 to 1280×1024 using the R-squared fitness function in the experiment. Pixel dimensions of the FLC-SLM used in the experiment are 1280×1024, where the macro-pixel size has been explored from 1 to 32.

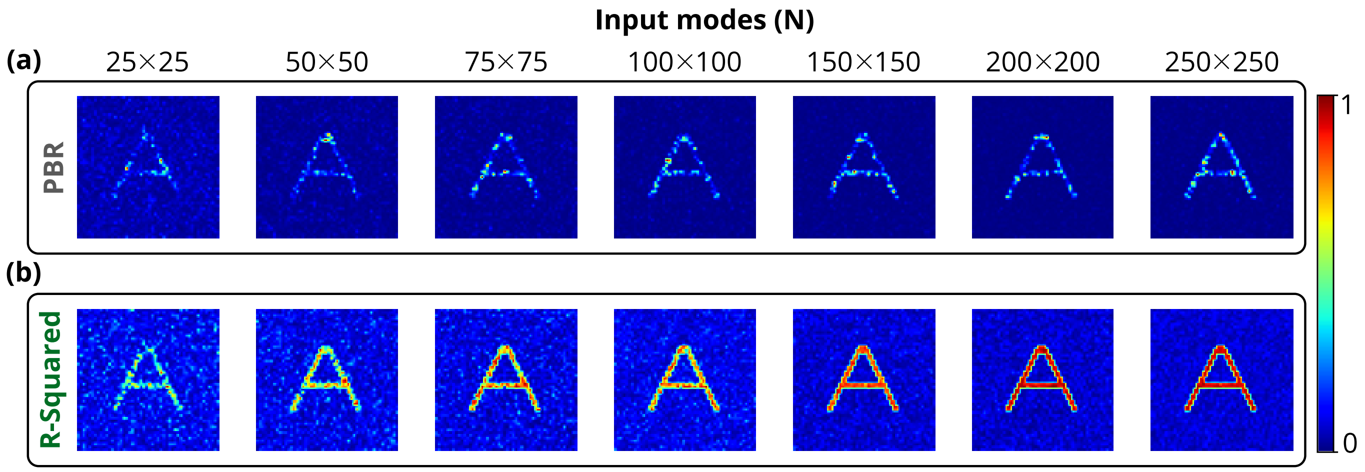

Figure S4: Effect of input modes number on the structure A. Simulation results for the different number of input modes and their effect on the structure 'A' using **(a).** PBR fitness function (top row) and **(b).** R-squared fitness function (bottom row).

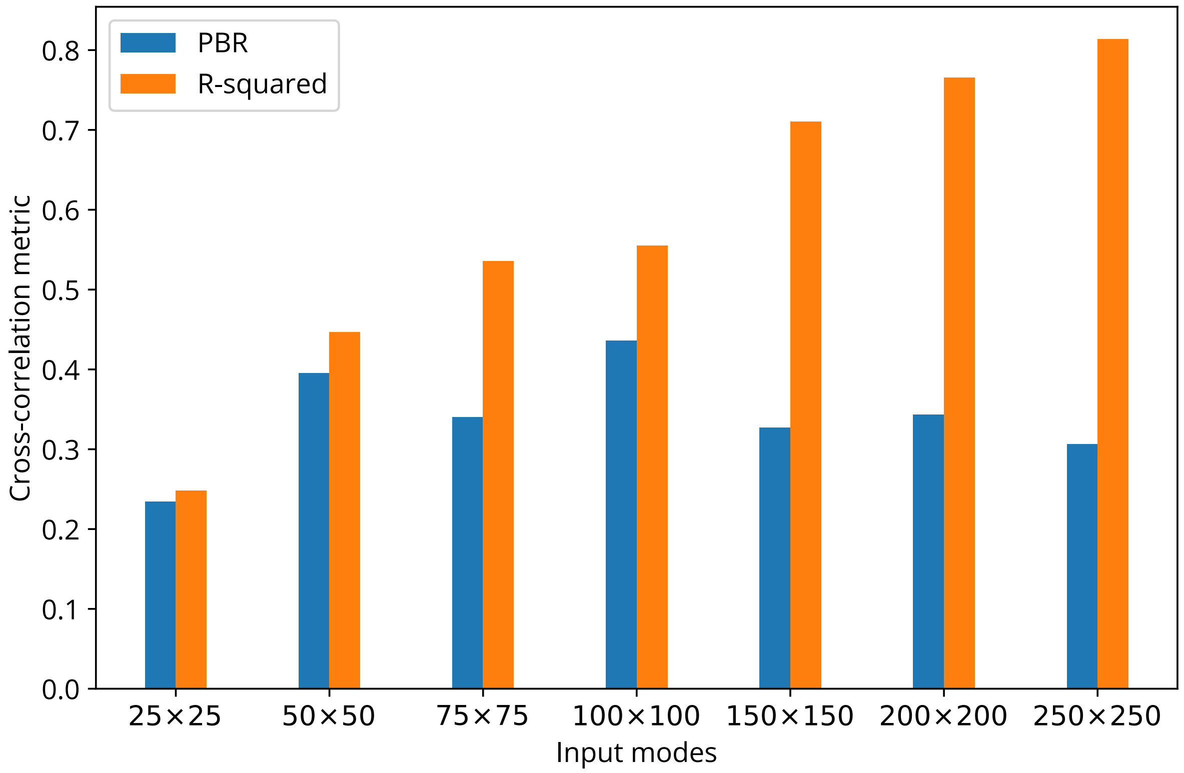

Figure S5: Effect of increasing the number of input modes on structure 'A'. Barplot shows the effect on cross-correlation metric while increasing the input modes using the PBR (blue bar) and R-squared fitness function (orange bar) for structure 'A'.

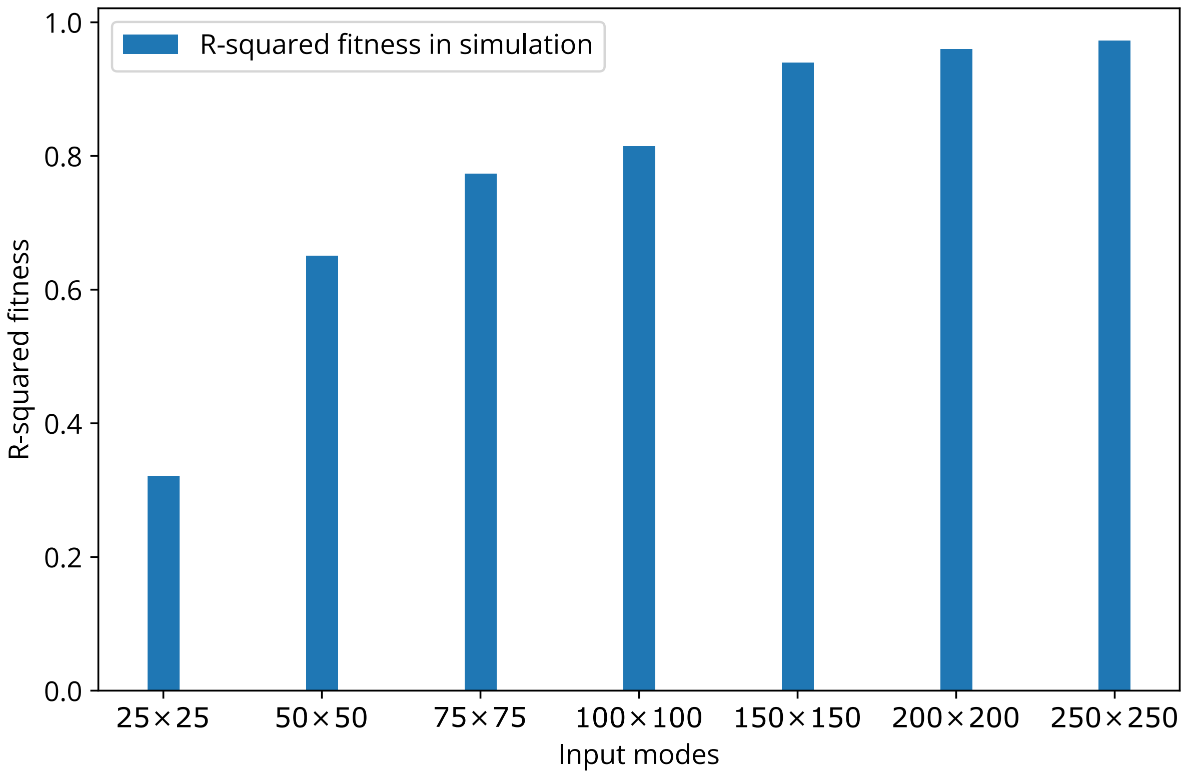

Figure S6: Effect of the number of input modes on structure 'A' using R-squared fitness function. shows the change in R-squared fitness value while increasing the input modes number for reconstructing structure 'A'.

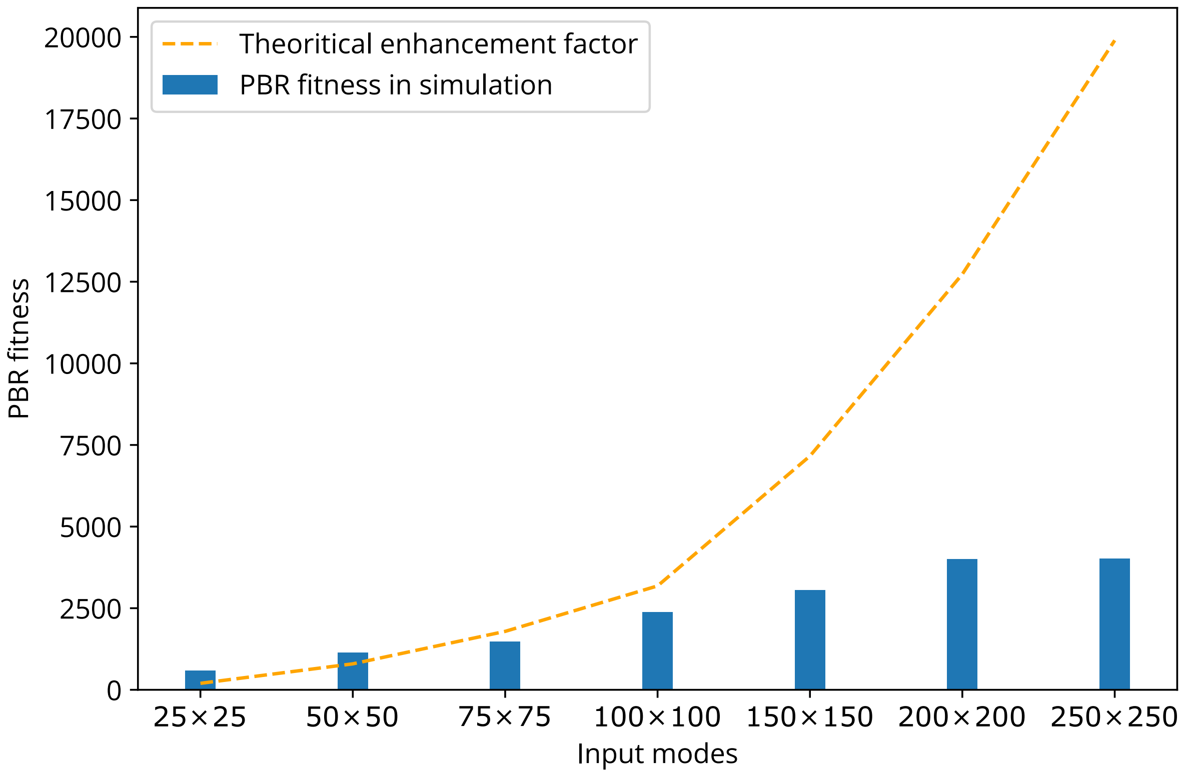

Figure S7: Effect of the number of input modes on structure 'A' using PBR fitness function. Simulation results show the different number of input modes and their PBR fitness value for structure 'A'.

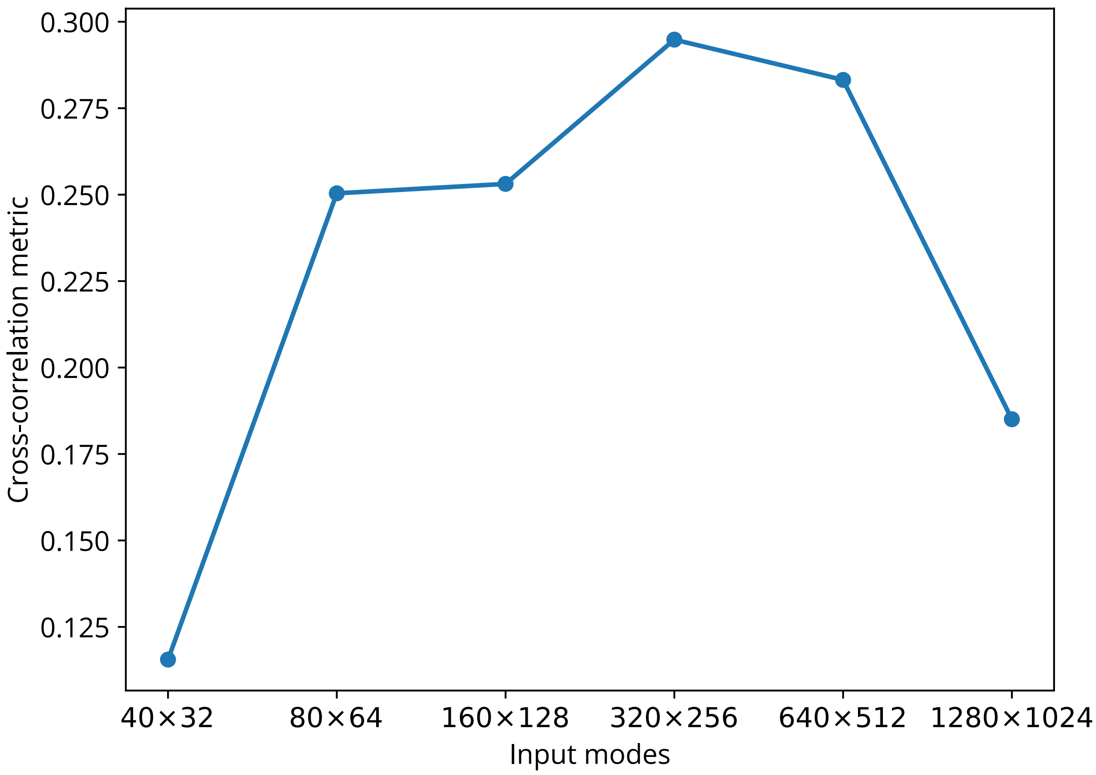

Figure S8: Effect of the number of input modes while reconstructing structure of letter 'A' using R-squared fitness function. Experimental results show the different number of input modes and their cross-correlation metric values for structure 'A'.

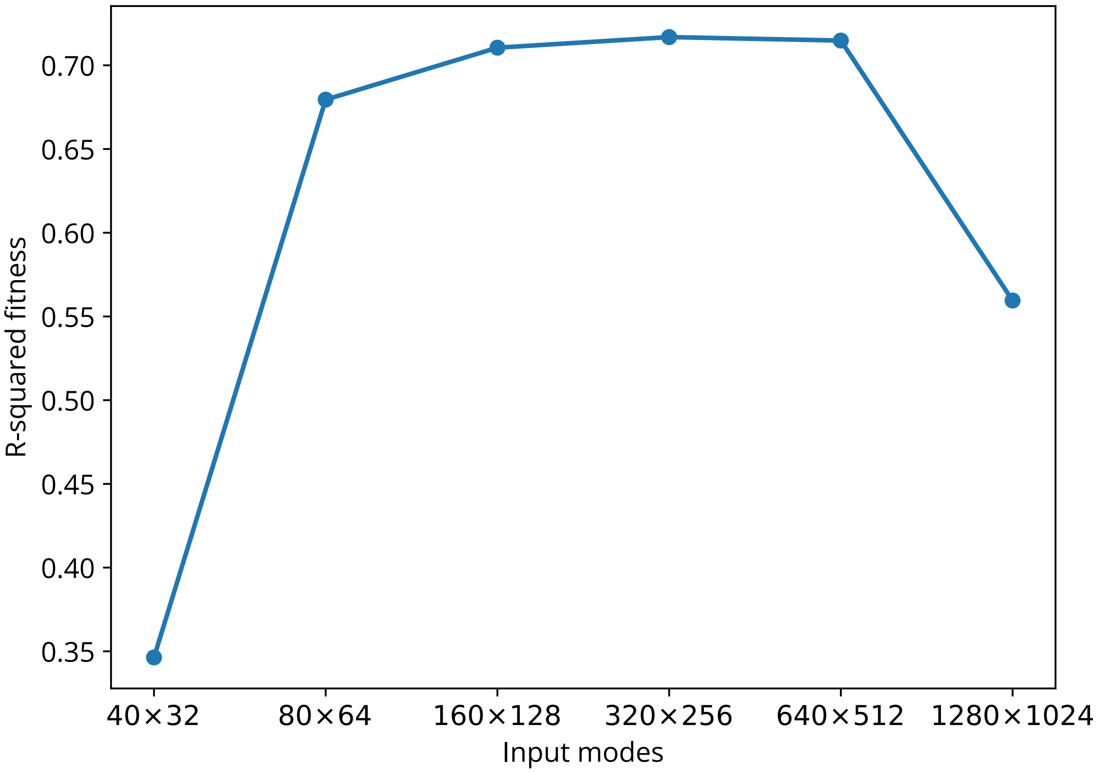

Figure S9: Effect of the number of input modes while reconstructing structure of letter 'A' using R-squared fitness function. Experimental results show the different number of input modes and their R-squared fitness value for structure 'A'.

S2.3 Standard deviation analysis of target and background pixels

The analysis of the standard deviation of the target intensity (T_σ) and the background intensity (B_σ) is shown in Figs. S10 and S11. These simulation and experimental results show that the PBR fitness function does not decrease the standard deviation of target pixels (T_σ) and background pixels (B_σ), which is significantly important to construct complex structures uniformly. To construct structure A uniformly, the R-squared fitness function decreases the standard deviation of the target pixels (T_σ) and the background pixels (B_σ).

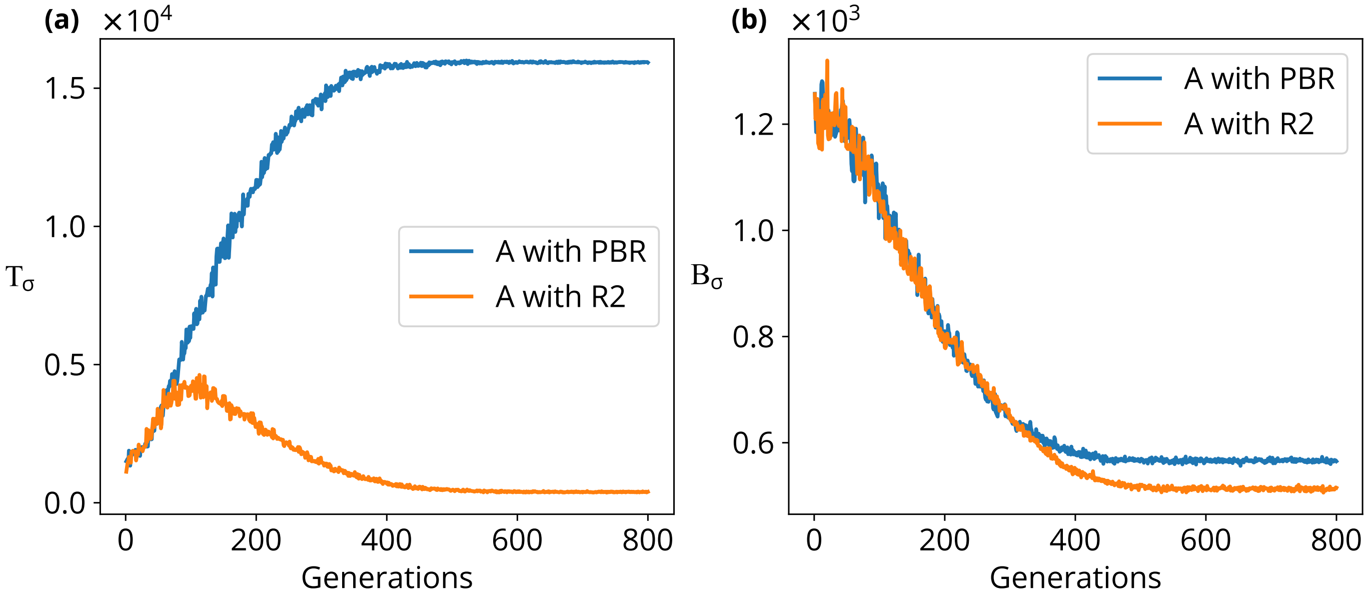

Figure S10: Simulation results for standard deviation of target and background pixels while constructing target structure 'A' using both R-squared and PBR fitness functions.. Figure **(a)** shows standard deviation of target pixels intensity (T_σ) and figure**(b)** shows standard deviation of the background pixels intensity (B_σ) analysis with generation.

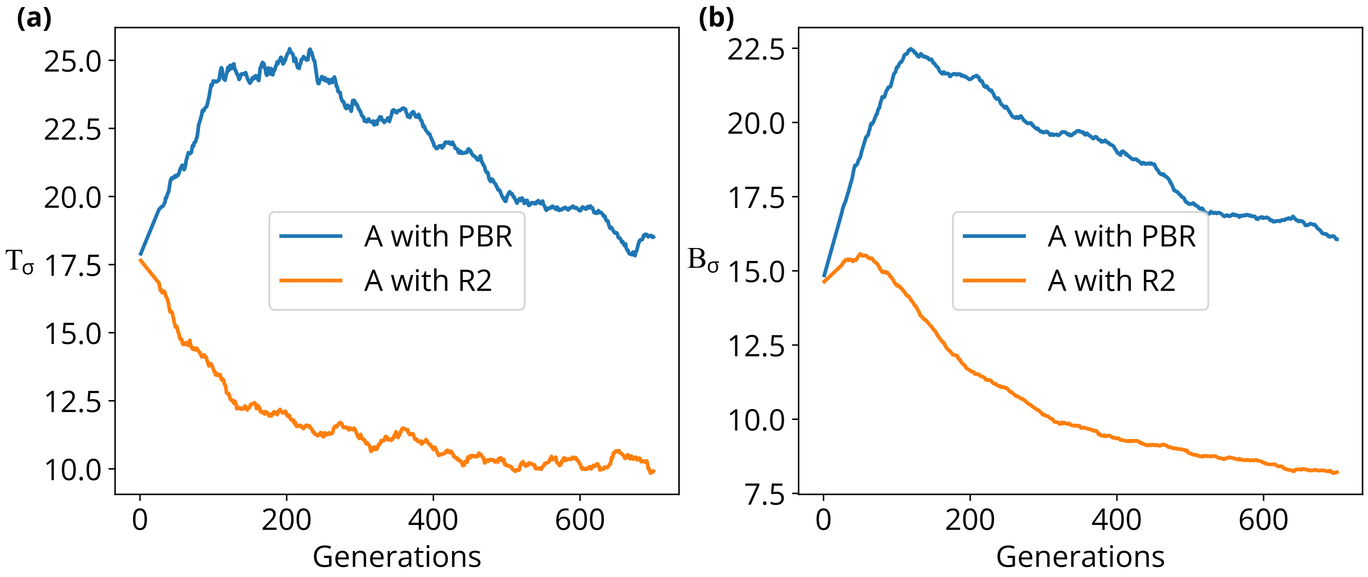

Figure S11: Experimental results for standard deviation of target and background pixels while constructing target structure 'A' using both R-squared and PBR fitness functions.. Figure **(a)** shows standard deviation of target pixels intensity (T_σ) and figure**(b)** shows standard deviation of the background pixels intensity (B_σ) analysis with generation.

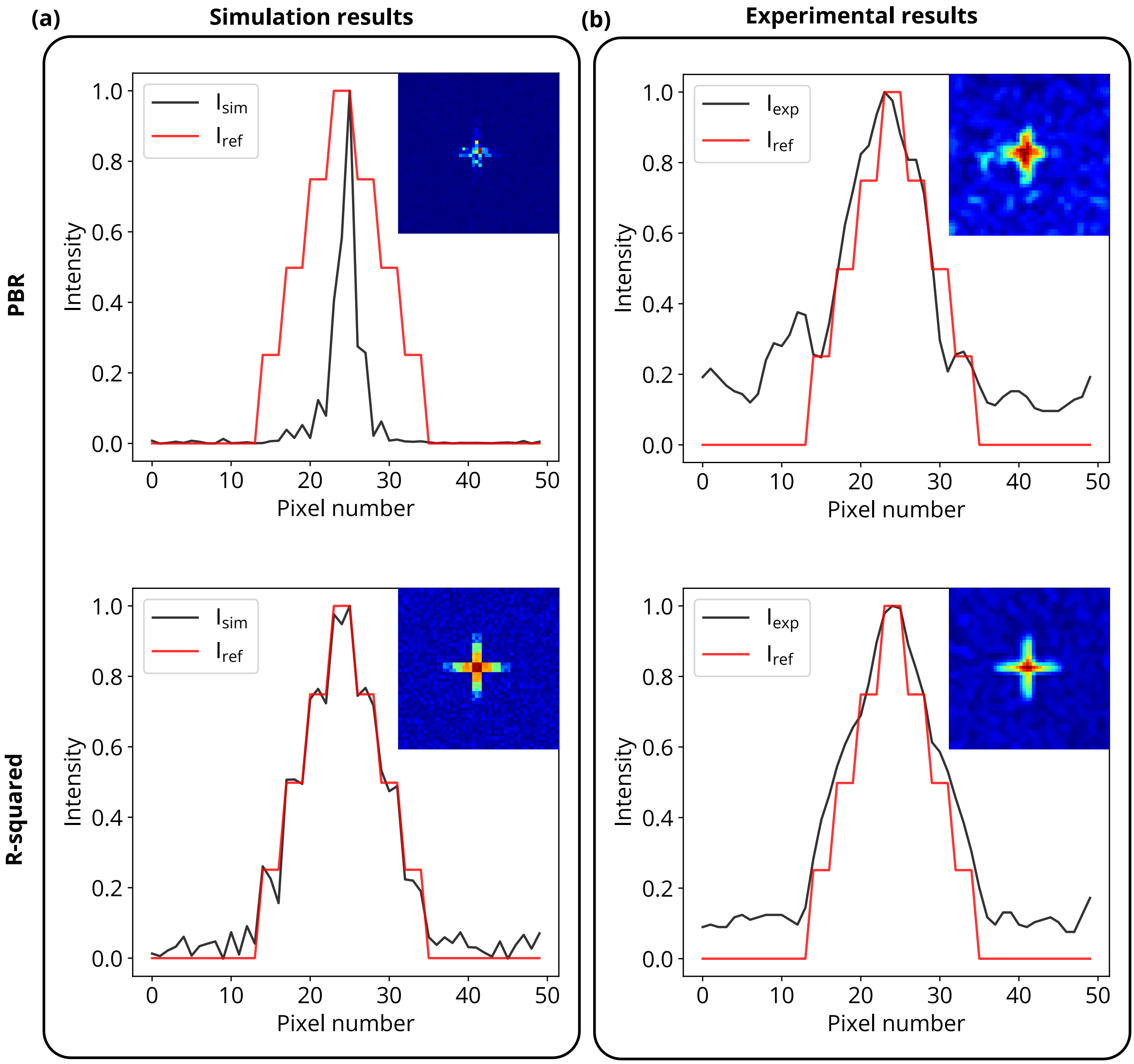

Figure S12: The 'plus' sign with gradient grayscale values and its line-plot analysis both in simulation and experimental results. Reference 'plus' sign structure image consisting of four different grayscale based target pixels and one grayscale level based background pixel. Figure (a) shows simulated results for the 'plus' sign structure and its line plot intensity using PBR and R-squared fitness functions. Figure (b) shows experimental results for the 'plus' sign structure and its line plot intensity using PBR and R-squared fitness functions.

S3 Calculation of output modes in binary phase FLC-SLM

The following equations prove that the conjugate phase does not change the intensity at the output mode. For illustration, we consider a 2x2 transmission matrix, t_{R_i} and t_{Im_i} are the real and imaginary parts, respectively, of each element t_i of the transmission matrix. A_n and θ are the amplitude and phase of the input mode, respectively.

$$\vec{E}_{out} = T\vec{E}_{in}\,, \qquad where\ T = \begin{pmatrix} t_{R_1} + i\,t_{Im_1} & t_{R_2} + i\,t_{Im_2} \\ t_{R_3} + i\,t_{Im_3} & t_{R_4} + i\,t_{Im_4} \end{pmatrix}\ and\ \vec{E}_{in} = \begin{pmatrix} A_n\,e^{i\,\theta_1} \\ A_n e^{i\,\theta_2} \end{pmatrix} \tag{S2}$$

For binary phase modulation of FLC-SLM, if $\theta_1 = 0$ and $\theta_2 = \pi$, then above equation can be written as;

$$\begin{aligned} \vec{E}_{out} &= \begin{pmatrix} t_{R_1} + i\,t_{Im_1} & t_{R_2} + i\,t_{Im_2} \\ t_{R_3} + i\,t_{Im_3} & t_{R_4} + i\,t_{Im_4} \end{pmatrix} \begin{pmatrix} A_n\,e^{i\,0} \\ A_n e^{i\,\pi} \end{pmatrix} \\ &= A_n \begin{pmatrix} t_{R_1} + i\,t_{Im_1} & t_{R_2} + i\,t_{Im_2} \\ t_{R_3} + i\,t_{Im_3} & t_{R_4} + i\,t_{Im_4} \end{pmatrix} \begin{pmatrix} 1 \\ -1 \end{pmatrix} \\ &= A_n \begin{pmatrix} (t_{R_1} - t_{R_2}) + i\,(t_{Im_1} - t_{Im_2}) \\ (t_{R_3} - t_{R_4}) + i\,(t_{Im_3} - t_{Im_4}) \end{pmatrix} \end{aligned} \tag{S3}$$

Now, I_{out} can be written as;

$$I_{out} = A_n \begin{pmatrix} \sqrt{(t_{R_1} - t_{R_2})^2 + (t_{Im_1} - t_{Im_2})^2} \\ \sqrt{(t_{R_3} - t_{R_4})^2 + (t_{Im_3} - t_{Im_4})^2} \end{pmatrix} \tag{S4}$$

Similarly, if $\theta_1 = \pi$ and $\theta_2 = 0$, then the above equation can be written as;

$$\begin{aligned} \vec{E}_{out} &= \begin{pmatrix} t_{R_1} + i\,t_{Im_1} & t_{R_2} + i\,t_{Im_2} \\ t_{R_3} + i\,t_{Im_3} & t_{R_4} + i\,t_{Im_4} \end{pmatrix} \begin{pmatrix} A_n\,e^{i\,\pi} \\ A_n e^{i\,0} \end{pmatrix} \\ &= A_n \begin{pmatrix} t_{R_1} + i\,t_{Im_1} & t_{R_2} + i\,t_{Im_2} \\ t_{R_3} + i\,t_{Im_3} & t_{R_4} + i\,t_{Im_4} \end{pmatrix} \begin{pmatrix} -1 \\ 1 \end{pmatrix} \\ &= A_n \begin{pmatrix} (t_{R_2} - t_{R_1}) + i\,(t_{Im_2} - t_{Im_1}) \\ (t_{R_4} - t_{R_3}) + i\,(t_{Im_4} - t_{Im_3}) \end{pmatrix} \end{aligned} \tag{S5}$$

Furthermore, I_{out} can be written as;

$$\begin{aligned} I_{out} &= A_n \begin{pmatrix} \sqrt{[-(t_{R_1} - t_{R_2})]^2 + [-(t_{Im_1} - t_{Im_2})]^2} \\ \sqrt{[-(t_{R_3} - t_{R_4})]^2 + [-(t_{Im_3} - t_{Im_4})]^2} \end{pmatrix} \\ &= A_n \begin{pmatrix} \sqrt{(t_{R_1} - t_{R_2})^2 + (t_{Im_1} - t_{Im_2})^2} \\ \sqrt{(t_{R_3} - t_{R_4})^2 + (t_{Im_3} - t_{Im_4})^2} \end{pmatrix} \end{aligned} \tag{S6}$$

This calculation also proves that, for binary-phase modulation, mutation rates 1% and 99% have an identical effect on a particular mask.

S4 Experimental details

S4.1 Photograph of the developed experimental setup with iterative binary phase modulation in FLC-SLM

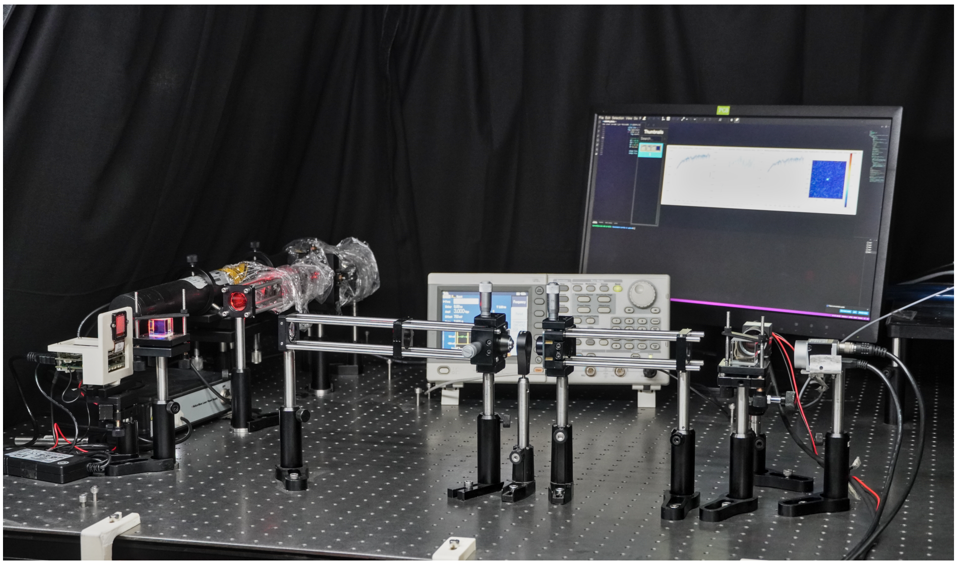

Figure S13: The photograph shows the experimental setup with various optical components. The light coming from the He-Ne laser (633 nm) is spatially filtered and passed on to the SLM, where it gets modulated, the reflected light from the SLM goes through a $4F$ setup and gets projected on the scattering media with the help of an objective lens. A second objective is placed behind the scattering media to image the plane on a camera that sends the feedback to the computer. Two cameras are placed on the two arms of the beam splitter such that they capture images from different planes simultaneously behind the scattering media. A function generator is used to synchronize the output signal from FLC-SLM and to trigger the two cameras.

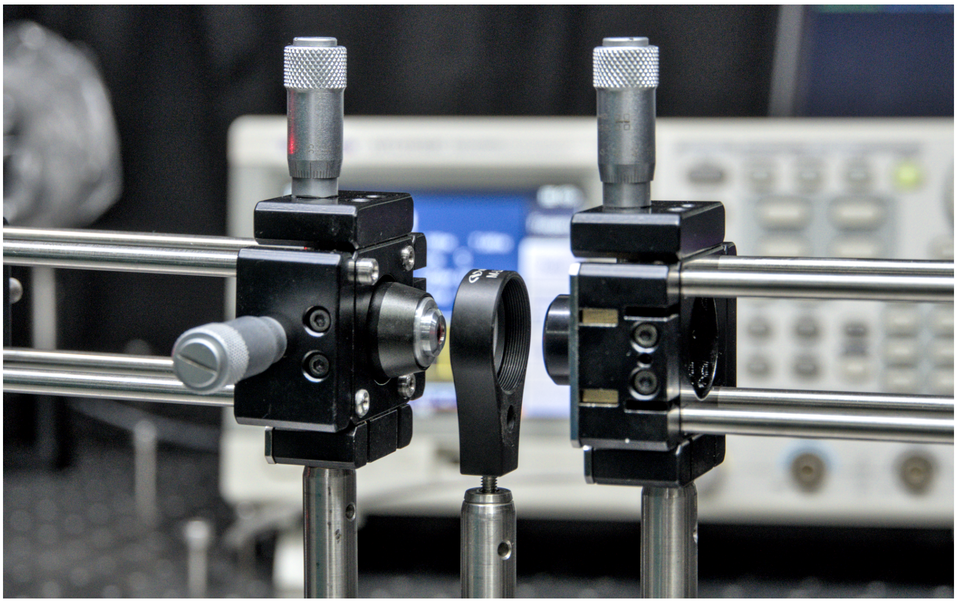

Figure S14: A close-up view of the scattering media mounted between two objective lenses. The first objective projects the modulated wavefront from the FLC-SLM on the scattering media, and the second objective images the plane behind the scattering media on a CMOS camera sensor.

S4.2 The process of synchronizing the cameras with the FLC-SLM

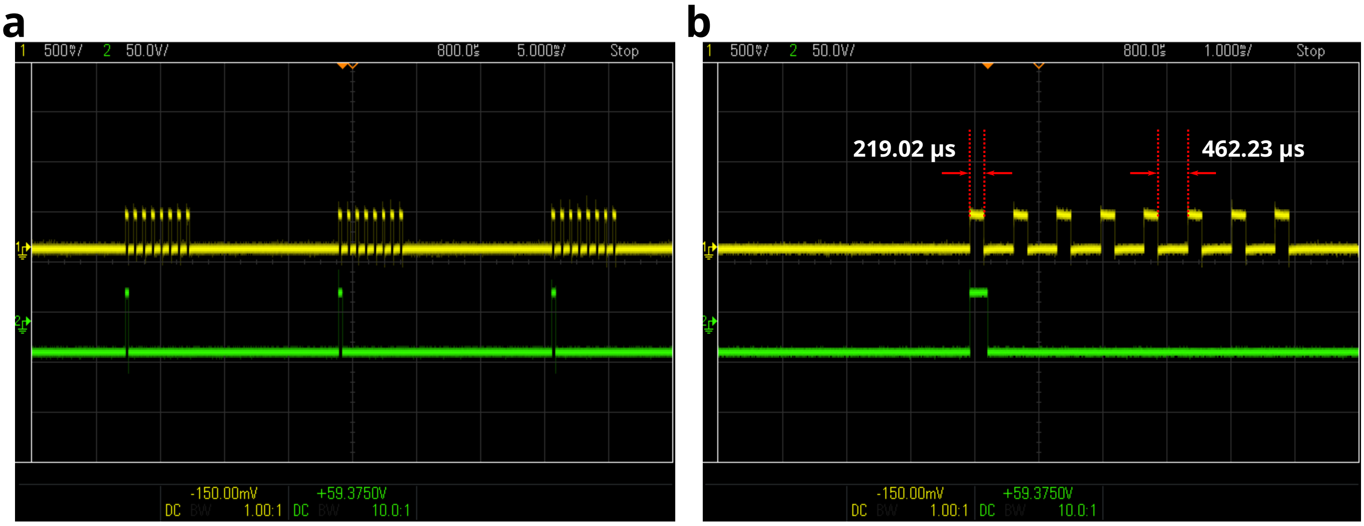

Figure S15: Figure explains the bit planes of the FLC-SLM. In the left image, the yellow signal (24 pulses) is from the SLM, and the green signal is one of the trigger signals generated by the function generator. In the right image, the 8 pulses represent 8 red channels bit planes, their pulse width, and resting time between the two consecutive pulses of the SLM out of the total bit planes of 24. The green pulse signal shows the pulse width of the generated trigger signal with the function generator that is used to trigger the cameras.

S4.3 Axial resolution of the experimental system

Additional experiments have been performed to measure the axial resolution of the system, and the results are provided in Fig. S16. The structures "A" and "O" are slightly overlapping when both are placed at back-to-back positions in two planes separated by 75 µm (Fig. S16(a)). The resolution of the system has been estimated with the objective lens (10X, 0.25 NA) at wavelength 633 nm and has been found to be 88.15 $\pm$2 µm, S16(e). A more complex volume imaging with multiple images in 3D volume is shown in Fig. S16(d). However, The overlapping completely disappeared when two planes are separated by 99.7 $\pm$ 2 µm (Fig. S16(f)).

S4.4 Working principle of FLC-SLM

Fig. S17 explains the working principle of FLC-SLM and PBS using the vector representation diagram. Here, $\vec{E}_{in}$ is the input linearly polarized light vector. The FLC-SLM works as a half-wave plate on $\vec{E}_{in}$. A binary phase mask is displayed on the FLC-SLM, where the black pixels represent phase 0 and the white pixels represent phase π. For

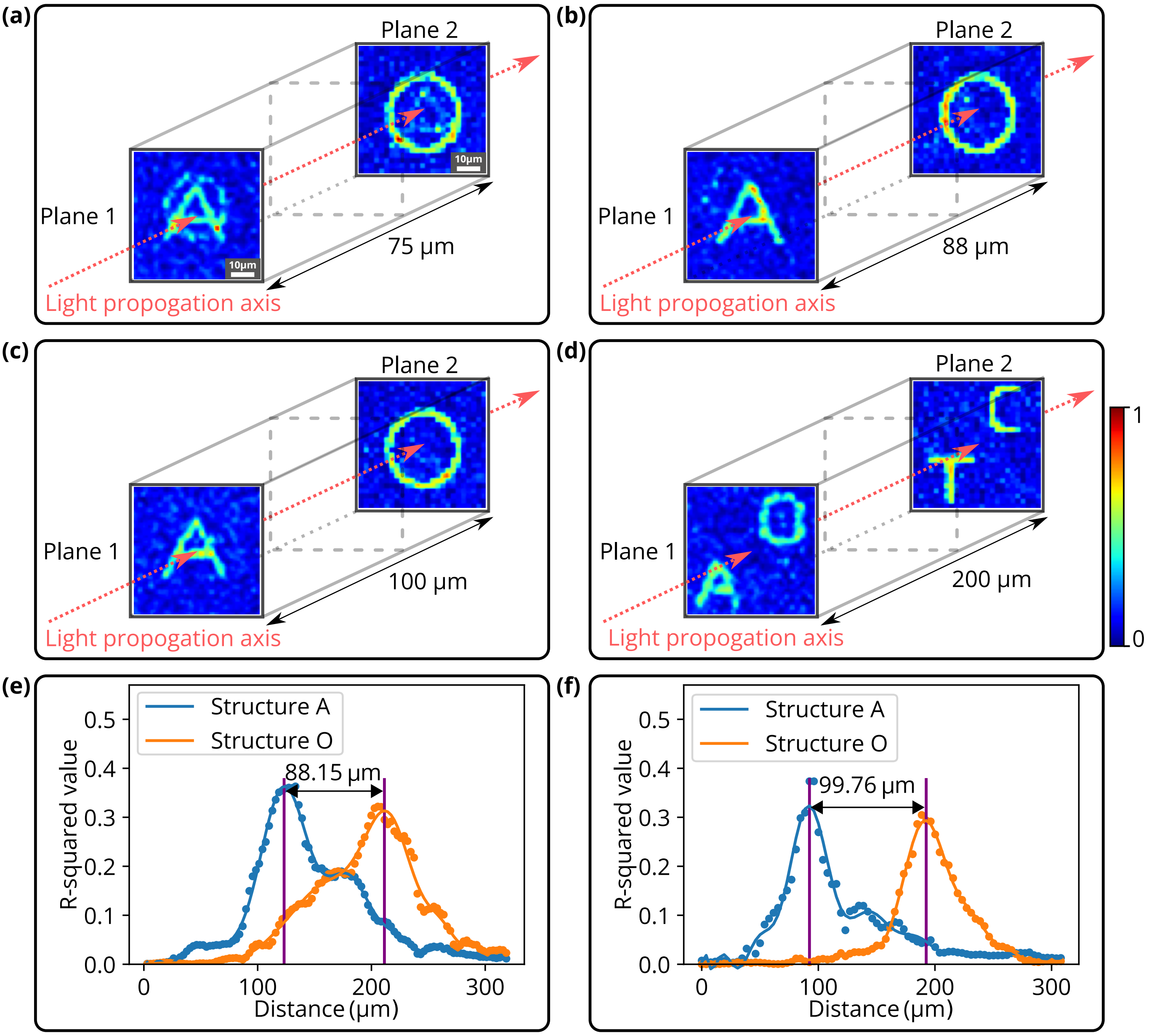

Figure S16: 3D structures formation at different depths. Where, **(a), (b), and (c)** show the formation of letters **A** and **O** at back to back planes separated by 75 µm, 88 µm, and 100 µm respectively. **(d)** shows the formation of multiple letters **A**, **O**, **T**, and **C** at two planes separated by 200 µm apart. **(e)** and **(f)** shows the fitness value vs lineplots along the optical axis in 3D volume for figure (b) and figure (c) respectively. All the experimental results have the same scale bar of 10 µm.

black pixels, the fast axis of the liquid crystal is aligned with $\hat{e}_1$, and for white pixels, it is aligned with $\hat{e}_2$. Thus, $\vec{E}_{in}$ is rotated by 2θ and converted into $\vec{E}_1$ and $\vec{E}_2$ for the black pixel and the white pixel, respectively. The polarization axis of the PBS transmission mode is set to a horizontal position. Thus, $\vec{E}_1$ and $\vec{E}_2$ are horizontally decomposed by PBS and the output light vectors $\vec{E}_{out1}$ (with phase 0) and $\vec{E}_{out2}$ (with phase π) are formed. The decomposed vectors $\vec{E}_{out1}$ and $\vec{E}_{out2}$ are π out of phase.

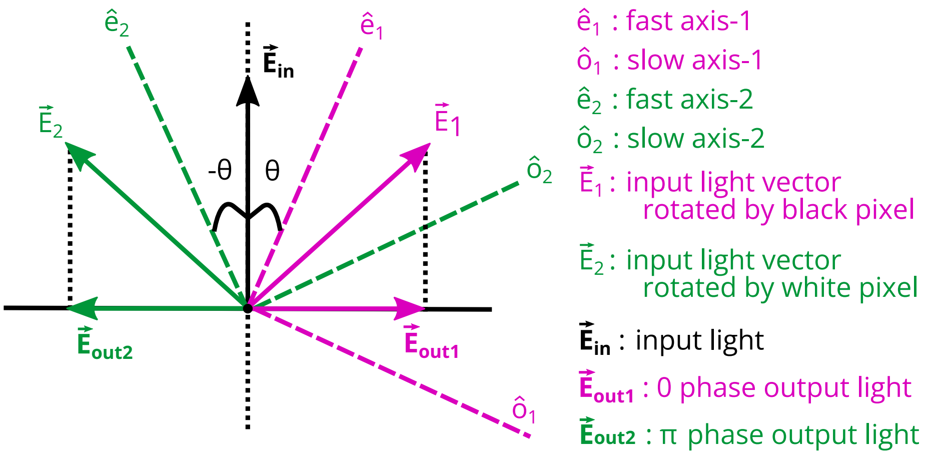

Figure S17: FLC-SLM working vector diagram.

S4.5 Noise analysis in experiments

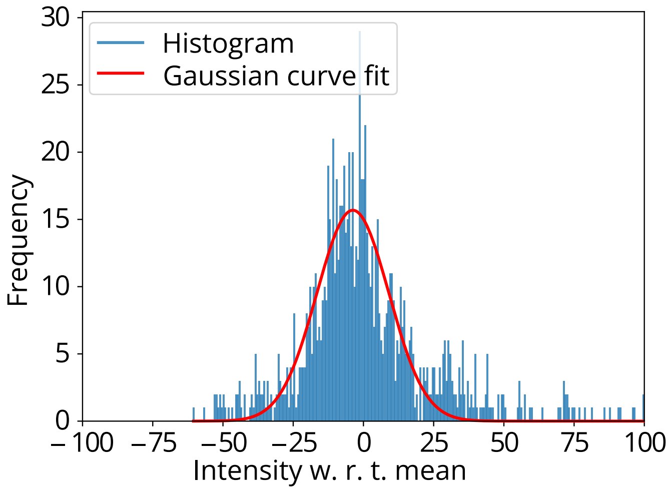

Figure S18: Noise profile in the experimental setup. The figure shows the intensity noise distribution in the setup. The calculated average noise in the experiment is $\sim 28.78\,\%$.

References

[1] Anderson, B. R., Gunawidjaja, R. & Eilers, H. Effect of experimental parameters on optimal transmission of light through opaque media. *Phys. Rev. A* **90**, 053826 (2014).

[2] Conkey, D. B., Brown, A. N., Caravaca-Aguirre, A. M. & Piestun, R. Genetic algorithm optimization for focusing through turbid media in noisy environments. *Opt. Express* **20**, 4840–4849 (2012).

[3] Fang, L., Zhang, X., Zuo, H. & Pang, L. Focusing light through random scattering media by four-element division algorithm. *Opt. Commun.* **407**, 301–310 (2018).

[4] Conkey, D. B. & Piestun, R. Color image projection through a strongly scattering wall. *Opt. Express* **20**, 27312–27318 (2012).

[5] Vellekoop, I. M. Feedback-based wavefront shaping. *Opt. Express* **23**, 12189–12206 (2015).

[6] Wan, L., Chen, Z., Huang, H. & Pu, J. Focusing light into desired patterns through turbid media by feedback-based wavefront shaping. *Applied Physics B* **122**, 204 (2016).

[7] Feng, Q., Zhang, B., Liu, Z., Lin, C. & Ding, Y. Research on intelligent algorithms for amplitude optimization of wavefront shaping. *Appl. Opt.* **56**, 3240–3244 (2017).

[8] Zhang, B. *et al.* Focusing light through strongly scattering media using genetic algorithm with SBR discriminant. *J. Opt.* **20**, 025601 (2017).
